# Linked magnolol dimer as a selective PPARγ agonist – Structure-based rational design, synthesis, and bioactivity evaluation

**DOI:** 10.1038/s41598-017-12628-5

**Published:** 2017-10-20

**Authors:** Dominik Dreier, Simone Latkolik, Lukas Rycek, Michael Schnürch, Andrea Dymáková, Atanas G. Atanasov, Angela Ladurner, Elke H. Heiss, Hermann Stuppner, Daniela Schuster, Marko D. Mihovilovic, Verena M. Dirsch

**Affiliations:** 1 Institute of Applied Synthetic Chemistry, TU Wien, Vienna, Austria; 2 Department of Pharmacognosy, University of Vienna, Vienna, Austria; 3 Department of Biochemical Sciences, Faculty of Pharmacy in Hradec Králové, Charles University, Hradec Králové, Czech Republic; 4 Institute of Genetics and Animal Breeding of the Polish Academy of Sciences, Jastrzebiec, Poland; 5 Institute of Pharmacy/Pharmacognosy and Center for Molecular Biosciences Innsbruck (CMBI), University of Innsbruck, Innsbruck, Austria; 6 Institute of Pharmacy/Pharmaceutical Chemistry and Center for Molecular Biosciences Innsbruck (CMBI), University of Innsbruck, Innsbruck, Austria

## Abstract

The nuclear receptors peroxisome proliferator-activated receptor γ (PPARγ) and its hetero-dimerization partner retinoid X receptor α (RXRα) are considered as drug targets in the treatment of diseases like the metabolic syndrome and diabetes mellitus type 2. Effort has been made to develop new agonists for PPARγ to obtain ligands with more favorable properties than currently used drugs. Magnolol was previously described as dual agonist of PPARγ and RXRα. Here we show the structure-based rational design of a linked magnolol dimer within the ligand binding domain of PPARγ and its synthesis. Furthermore, we evaluated its binding properties and functionality as a PPARγ agonist *in vitro* with the purified PPARγ ligand binding domain (LBD) and in a cell-based nuclear receptor transactivation model in HEK293 cells. We determined the synthesized magnolol dimer to bind with much higher affinity to the purified PPARγ ligand binding domain than magnolol (*K*_i_ values of 5.03 and 64.42 nM, respectively). Regarding their potency to transactivate a PPARγ-dependent luciferase gene both compounds were equally effective. This is likely due to the PPARγ specificity of the newly designed magnolol dimer and lack of RXRα-driven transactivation activity by this dimeric compound.

## Introduction

The nuclear receptor peroxisome proliferator-activated receptor γ (PPARγ) is a central signal integrator in maintaining lipid and glucose homeostasis and is used as clinical target to treat diseases such as type 2 diabetes mellitus and the metabolic syndrome^1,2^. Furthermore, PPARγ has implications in the treatment of inflammatory and cardiovascular diseases^3^. The receptor is a member of a superfamily of ligand-activated transcription factors that controls lipid and glucose metabolism in various tissues such as adipose tissue, heart, lung, kidney, liver, large intestine, and macrophages. Ligand binding to PPARs leads to the formation of heterodimers with retinoid X receptors (RXR) to regulate transcription of target genes by binding specific response elements within their promoter regions^4^. Currently used full PPARγ agonists in clinics (thiazolidinediones, e.g. pioglitazone) are effective but trigger serious side effects such as formation of edema and weight gain. It has been shown that partial PPARγ agonists eliciting submaximal activation produce fewer side effects keeping beneficial effects for clinical treatments^5^.

Within a transdisciplinary research program^6^ aiming at the identification of novel natural products displaying anti-inflammatory activity^7^, we recently identified magnolol and honokiol as interesting structures displaying pharmacological activity as partial agonists for PPARγ^8,9^. Both, magnolol and honokiol act additionally as ligands for its hetero-dimerization partner RXRα^10,11^.

This work focused on the ambition to further exploit the biaryl-system of magnolol and honokiol in order to create selective PPARγ ligands^12^ with no concomitant effect on RXRα. We made use of the recent report on the co-crystallization of magnolol with the ligand binding domains of RXRα and PPARγ^10^. Whereas two molecules of magnolol are positioned in rather close spatial proximity within the human PPARγ receptor ligand binding domain (LBD), only one magnolol molecule binds to the LBD of RXRα. This prompted us to hypothesize that a molecule combining the structural features of two magnolol entities tethered within a single compound could potentially fit only into the ligand binding domain of PPARγ in a very selective way. The aim of this study was therefore to design, synthesize, and evaluate the impact of a magnolol-derived dimer on PPARγ and RXRα activity.

## Results

### Computational design of the magnolol dimer

**Figure 1:**
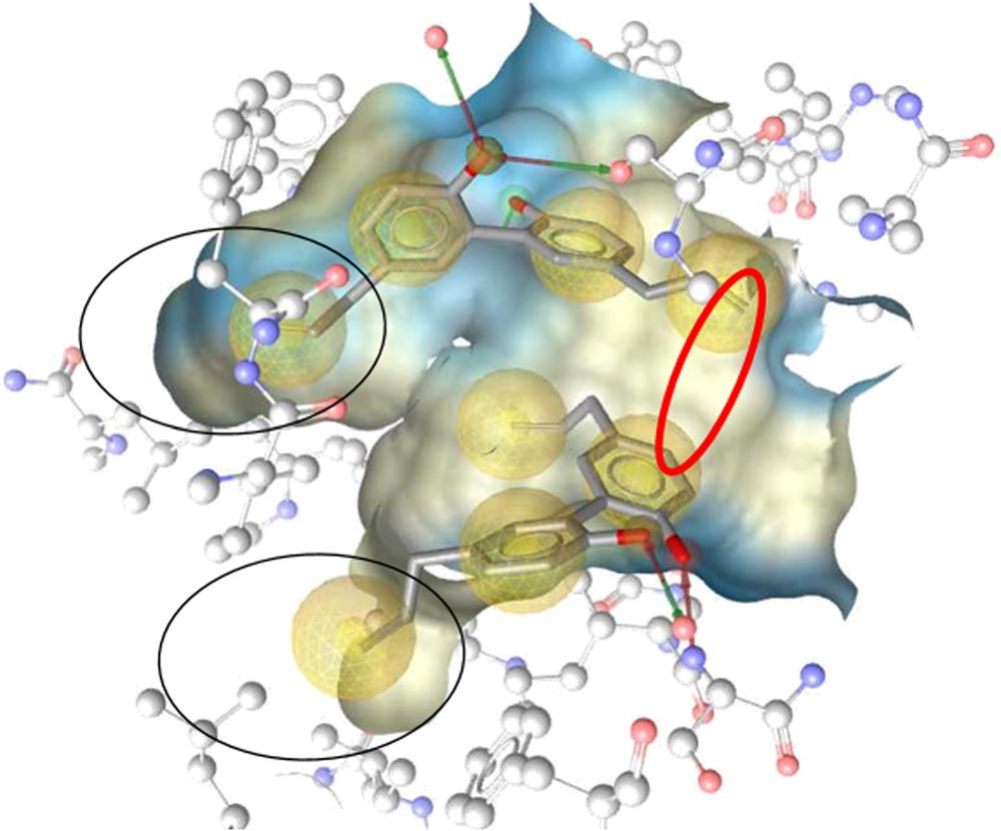
Two molecules magnolol co-crystallized within the ligand binding domain of PPARγ. Chemical protein-ligand interactions are indicated as yellow spheres (hydrophobic contacts) and arrows (hydrogen bonds). The shape of the binding site is color-coded by aggregated hydrophilicity (blue)/lipophilicity (grey). Black circles indicate side chains, where the molecules could not be linked due to steric reasons in the binding site. The red circle highlights the position for which the linker chain was designed.

When co-crystallizing magnolol with human PPARγ (PDB entry 3r5n), Zhang *et al*. observed two magnolol molecules bound to the receptor ligand binding site (Fig. 1)^10^. An inspection of the 3D structure of the ligand binding domain looked for positions at which the two magnolol molecules could be linked together. First, we searched for atoms in both molecules that are preferably close to each other. An alkyl linker was manually designed so that the newly formed bonds had angles allowing for sp^2^ or sp^3^ hybridization of the attached atoms. The designed linker involved an aromatic ring and the terminal unsaturated allylic chain of the two molecules, respectively (red circle in Fig. 1).

**Figure 2:**
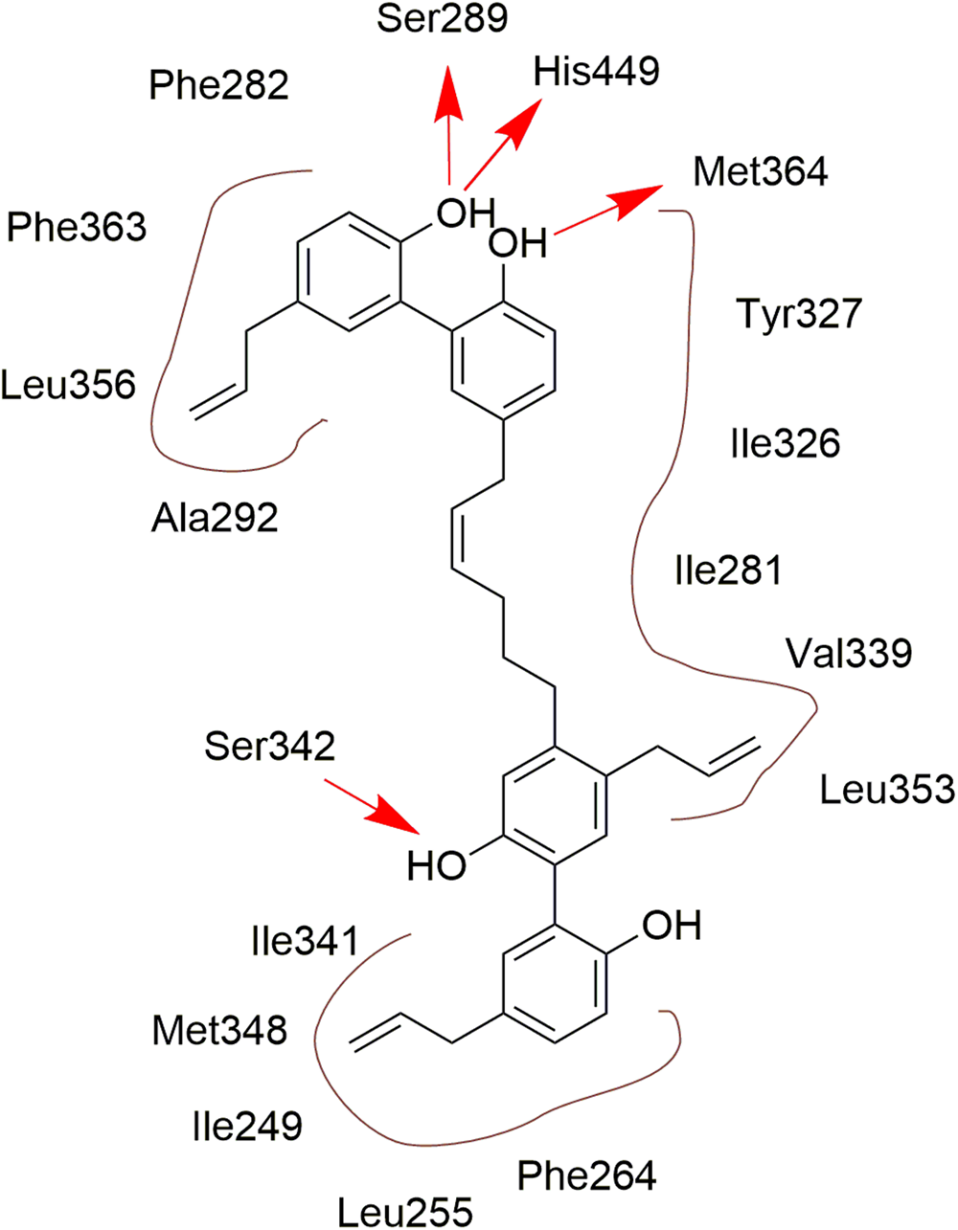
The designed magnolol dimer (**1**) with its predicted protein-ligand interactions after energy minimization in the PPARγ ligand binding domain. Hydrophobic contacts are indicated as brown contour lines, hydrogen bond interactions as red arrows.

The linker was computationally designed directly within the 3D ligand binding domain step-by-step by growing the side chain starting from one molecule into the direction of the other molecule. To stabilize the 3D orientations of the final dimer, double bonds were included, where a specific *E*- or *Z*-geometry was required. The proposed dimer was finally energetically minimized within the binding site to simulate if it would retain the crucial protein-ligand interactions of the initial two magnolol molecules (Fig. 2).

**Figure 3:**
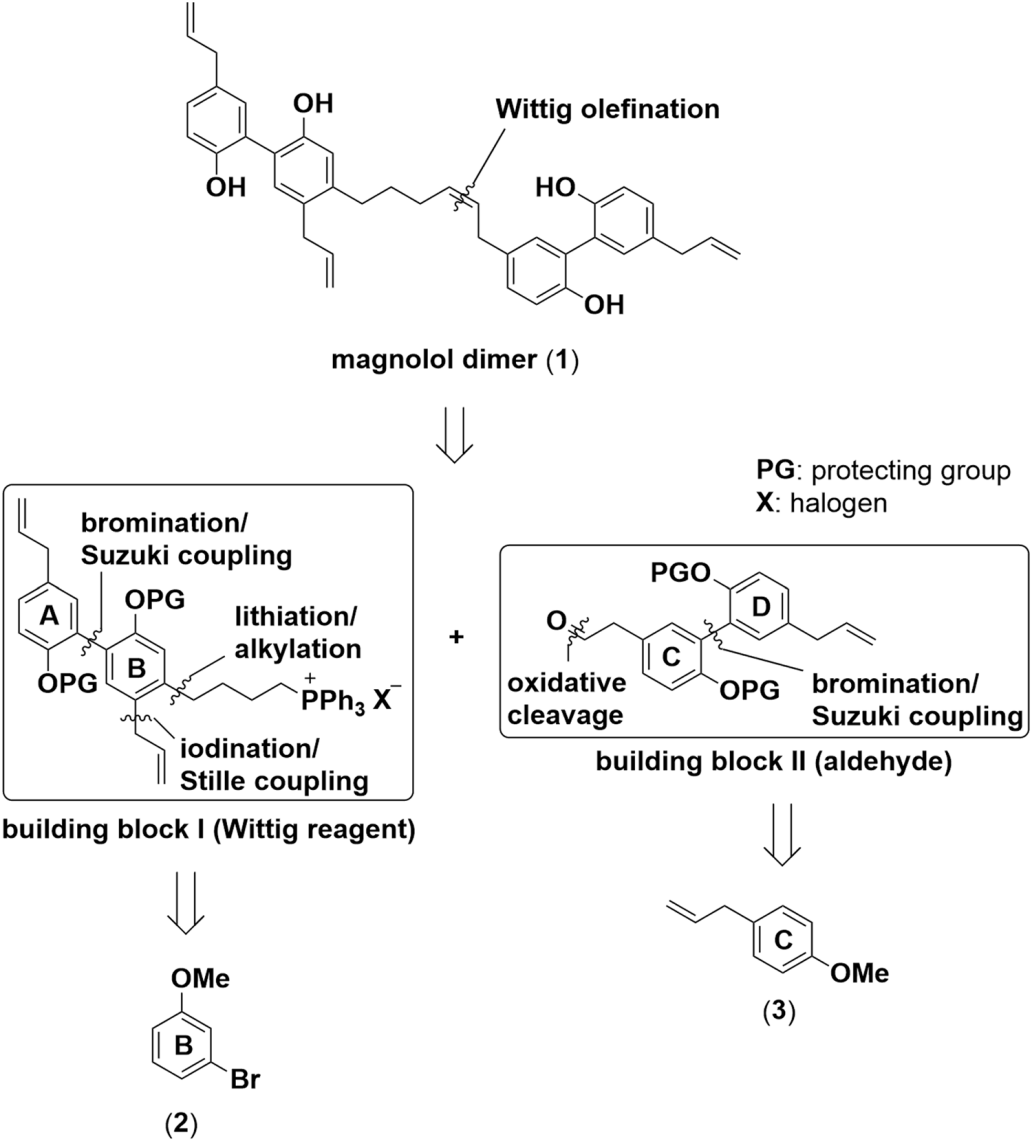
Retrosynthetic analysis of magnolol dimer (**1**) reveals the two key intermediates **I** and **II**, which can be synthesized from commercially available 1-bromo-3-methoxybenzene (**2**) and 1-allyl-4-methoxybenzene (**3**).

The structural analysis of the target molecule (magnolol dimer (**1**), see Fig. 3) revealed two biphenylic motifs, decorated with four phenolic hydroxyl groups and four allylic domains. One of the allylic domains is part of the linkage between the two individual magnolol motifs and the structural restrictions of the binding site require this double bond to exhibit *Z*-configuration. Therefore, we envisioned construction of this double bond by Wittig olefination using non-stabilized substrates to establish the desired configuration at the double bond. We decided to place the phosphonium (Wittig) salt on building block **I** and the aldehyde on building block **II** as this results in a shorter overall sequence. The biaryl moieties of both key intermediates **I** and **II** were intended to be constructed by metal catalyzed cross-coupling reactions using properly functionalized estragoles (junction of ring A with B and ring C with D). The tetra-substitution of ring B could be achieved starting from commercially available 1-bromo-3-methoxybenzene (**2**) by several consecutive steps involving alkylation, halogenations and regioselective cross-coupling reactions in a straight-forward fashion.

**Figure 4:**
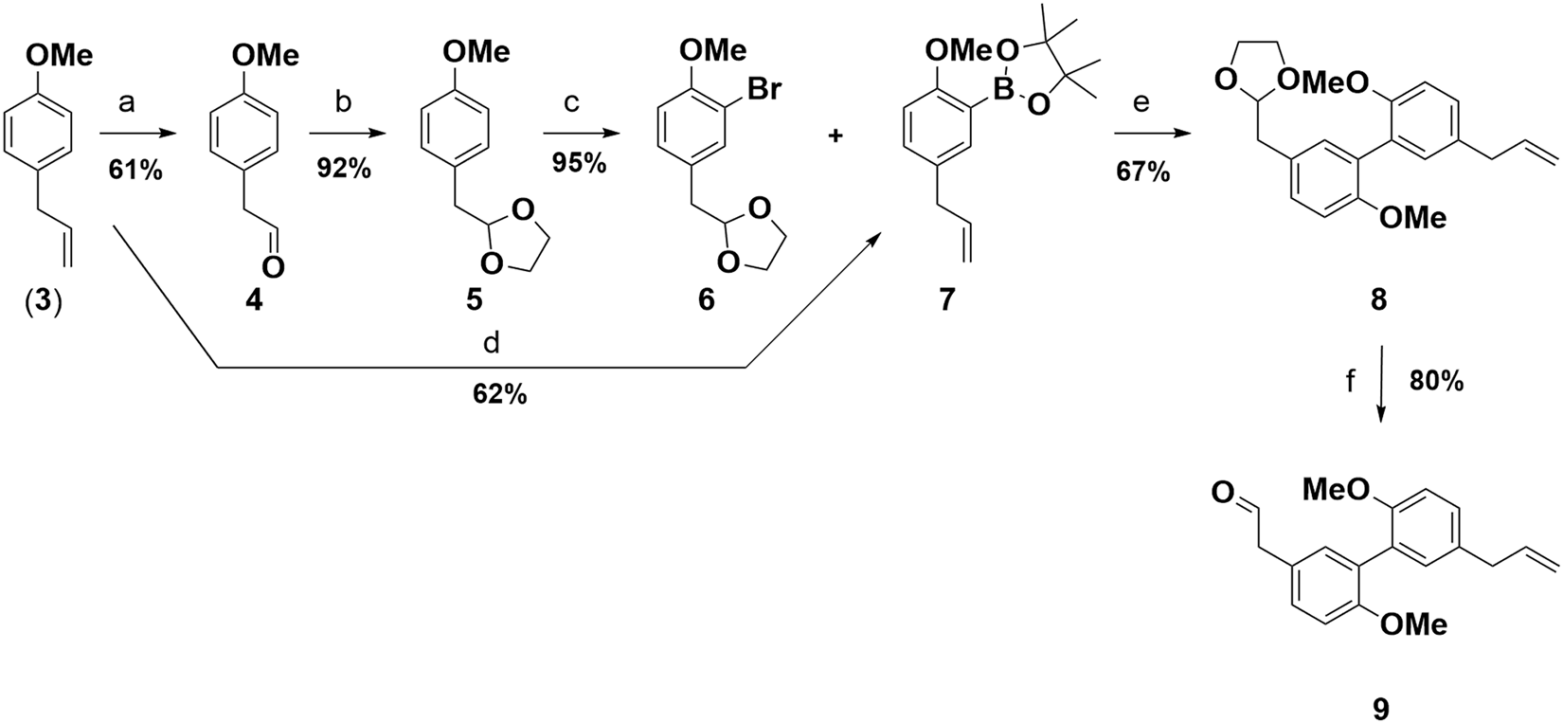
Preparation of key intermediate **9**: (**a**) OsO_4_, NaIO_4_, THF/H_2_O (1/1), 0 °C – rt; (**b**) ethylene glycol, PTSA∙H_2_O, toluene, reflux; (**c**) NBS, MeCN, 0 °C; (**d**) *s*-BuLi, TMEDA, THF, −70 °C – rt, then bis(pinacolato)diboron, rt; (**e**) Pd_2_(dba)_3_, SPhos, KF, THF/H_2_O (10/1), reflux; (**f**) HCl, µW, 120 °C.

Oxidative cleavage of 1-allyl-4-methoxybenzene (**3**) under Lemieux-Johnson conditions^13^ provided aldehyde **4** in 61%. Attempts to directly brominate **4** *via* electrophilic substitution failed due to limited stability of **4**; the labile aldehyde motif led to decomposition or oxidation to the corresponding carboxylic acid. Protection of the aldehyde with ethylene glycol under acidic conditions proceeded smoothly and furnished acetal **5** in 92%. Subsequent bromination with NBS afforded desired bromide **6** in 95%. Synthesis of coupling partner **7** started with *ortho* directed lithiation of 1-allyl-4-methoxybenzene (**3**), exploiting the coordinating ability of the methoxy group^14^. Subsequent treatment with bis(pinacolato)diboron yielded boronic acid ester **7** ^15,16^. For the following Suzuki coupling careful selection of reaction conditions was necessary in order to avoid isomerization of the allylic double bond to the thermodynamically more stable styrene. Employing conditions from Denton *et al*.^17^ on our reaction system turned out to promote Suzuki coupling and only trace amounts of the isomerized side product were detected. Biphenylic acetal **8** was obtained in 67%. Deprotection of the acetal **8** under acidic conditions led to partial decomposition of the material as well as to the isomerization of the double bond. Optimized reaction conditions under microwave irradiation gave aldehyde **9** in 80% yield while isomerization of the olefin was kept at a minimum (Fig. 4).

**Figure 5:**
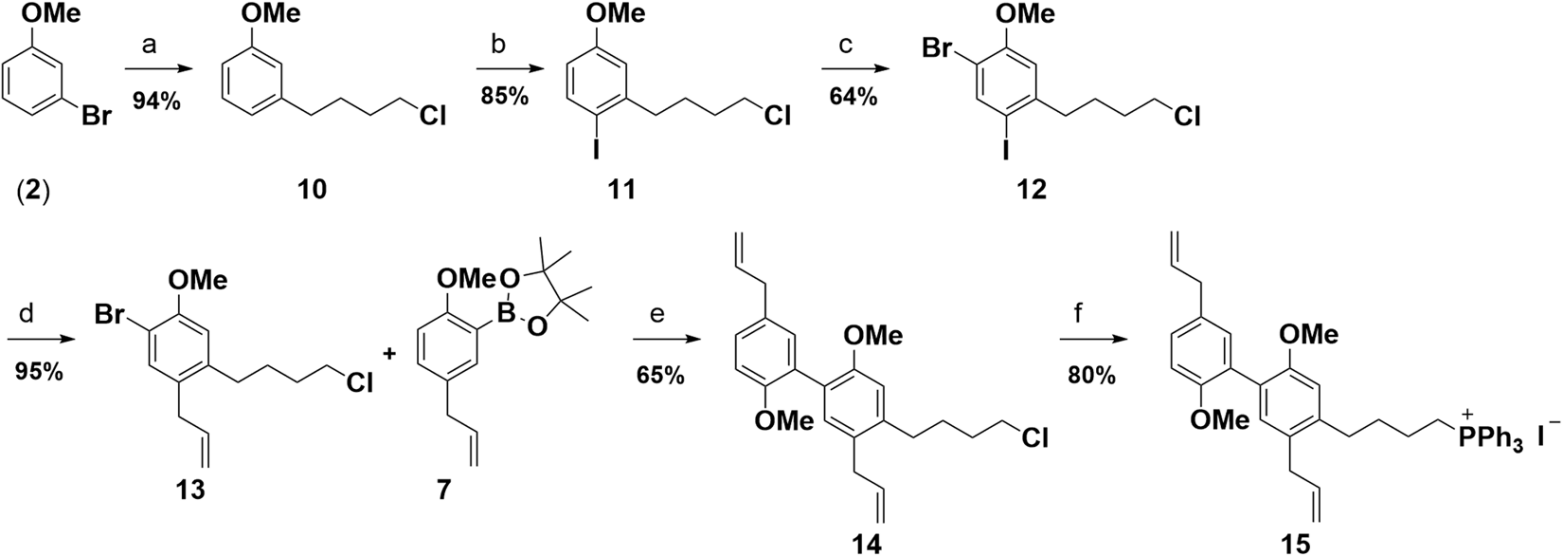
Preparation of key intermediate **15**: (**a**) *t*-BuLi, THF, -78 °C, then 1-bromo-4-chlorobutane, −78 °C – rt; (**b**) I_2_, HgO, Ac_2_O, DCM, reflux; (**c**) NBS, silica gel, BHT, protected from light, MeCN, −10 °C; (**d**) allyltributylstannane, Pd(PPh_3_)_4_, DMF, reflux; (**e**) Pd_2_(dba)_3_, SPhos, *t*-BuOK, THF, reflux; (**f**) NaI, PPh_3_, EtOAc, reflux.

Preparation of Wittig salt fragment **I** was initiated upon treatment of 1-bromo-3-methoxybenzene (**2**) with 2 equiv. of *t*-BuLi to afford the lithiated species cleanly, followed by reaction with 1-bromo-4-chlorobutane providing the alkylated product **10** in excellent 94% yield^18^. Subsequent iodination of **10** proceeded smoothly and *para* substituted iodoanisole **11** was obtained solely^19^. Intermediate **11** was further brominated with NBS requiring optimized reaction conditions^20^ leading to compound **12** in an isolated yield of 64%. Stille coupling of **12** with allyltributylstannane proceeded selectively to the more reactive iodine position and afforded compound **13** in excellent 95% yield^21^. Subsequently, **13** was subjected to Suzuki coupling with **7**. To our surprise, applying the identical condition as used previously on the Suzuki coupling of **6** with **7** led mainly to formation of the coupled product with isomerized double bonds. Consequently, optimization of the reaction conditions had to be re-visited and the problem was solved by using *t*-BuOK as the base in refluxing THF. Treatment of biphenylic chloride **14** with sodium iodide and triphenylphosphine provided Wittig salt **15** in 80% (Fig. 5)^22^.

**Figure 6:**
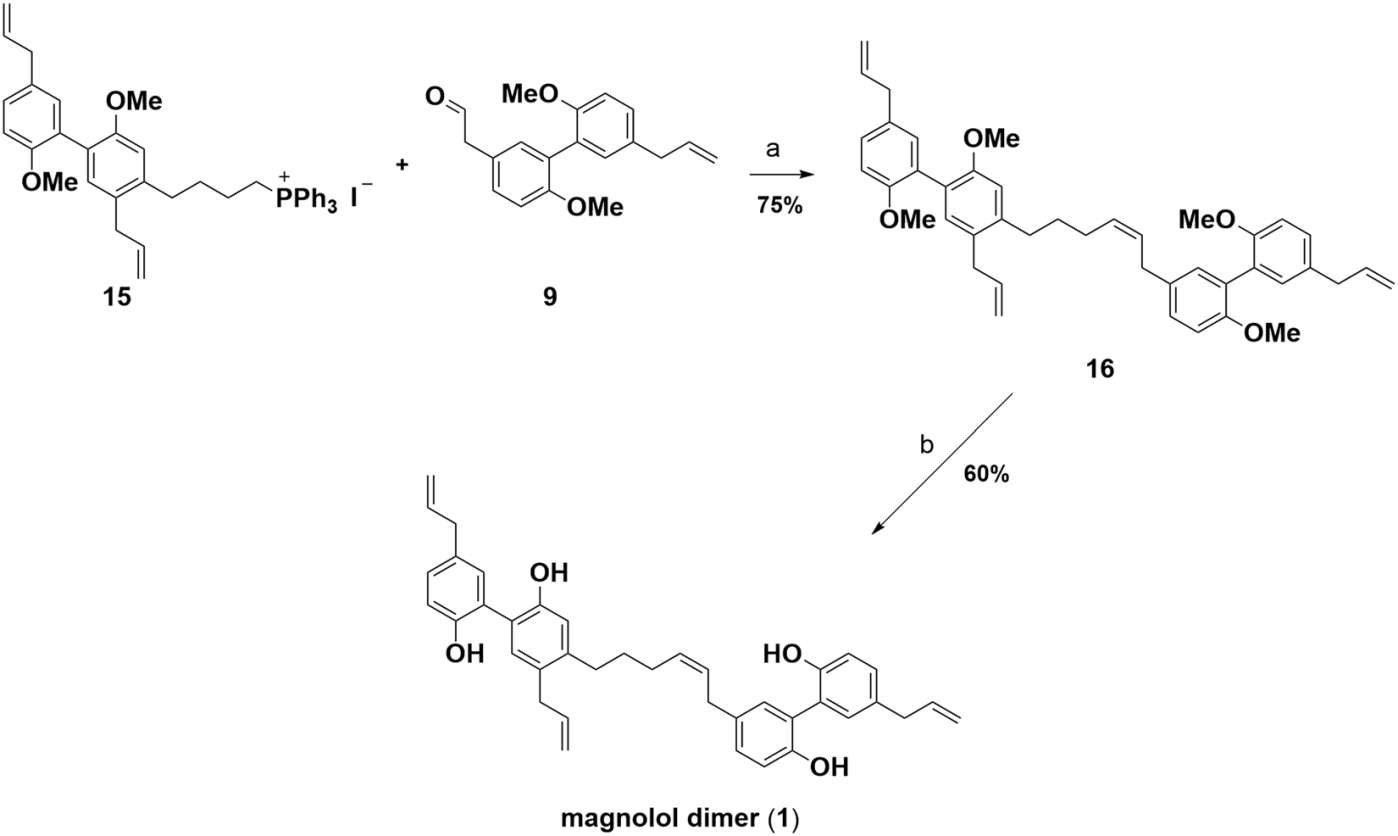
Preparation of the target compound, magnolol dimer (**1**): (**a**) KHMDS, ethyl ether, 0 °C – rt, then aldehyde, −55 °C – rt; (**b**) BBr_3_∙S(CH_3_)_2_, 1,2-dichloroethane, reflux.

Wittig reagent **15** was finally deprotonated with KHMDS in dry diethyl ether and reaction with aldehyde **9** afforded selectively *Z*-isomer **16** in an isolated yield of 75%^23^. Partial isomerization of the terminal double bonds was encountered under the reaction conditions employed leading to an inseparable mixture of products in >87% purity in favor of the expected structure. Global demethylation was carried out in refluxing 1,2-dichlorethane with BBr_3_ dimethyl sulfide complex resulting in formation of the target compound, magnolol dimer (**1**) (Fig. 6)^24^. After column chromatography no detectable quantities of isomerized side products were found in the isolated material by NMR. In summary, we successfully synthesized magnolol dimer (**1**) in a convergent multi-step route with an overall yield of 11.4% over the longest linear sequence; details on the synthesis are compiled in the Supplementary Information.

### Bioactivity evaluation of magnolol dimer (1) as PPARγ agonist

**Figure 7:**
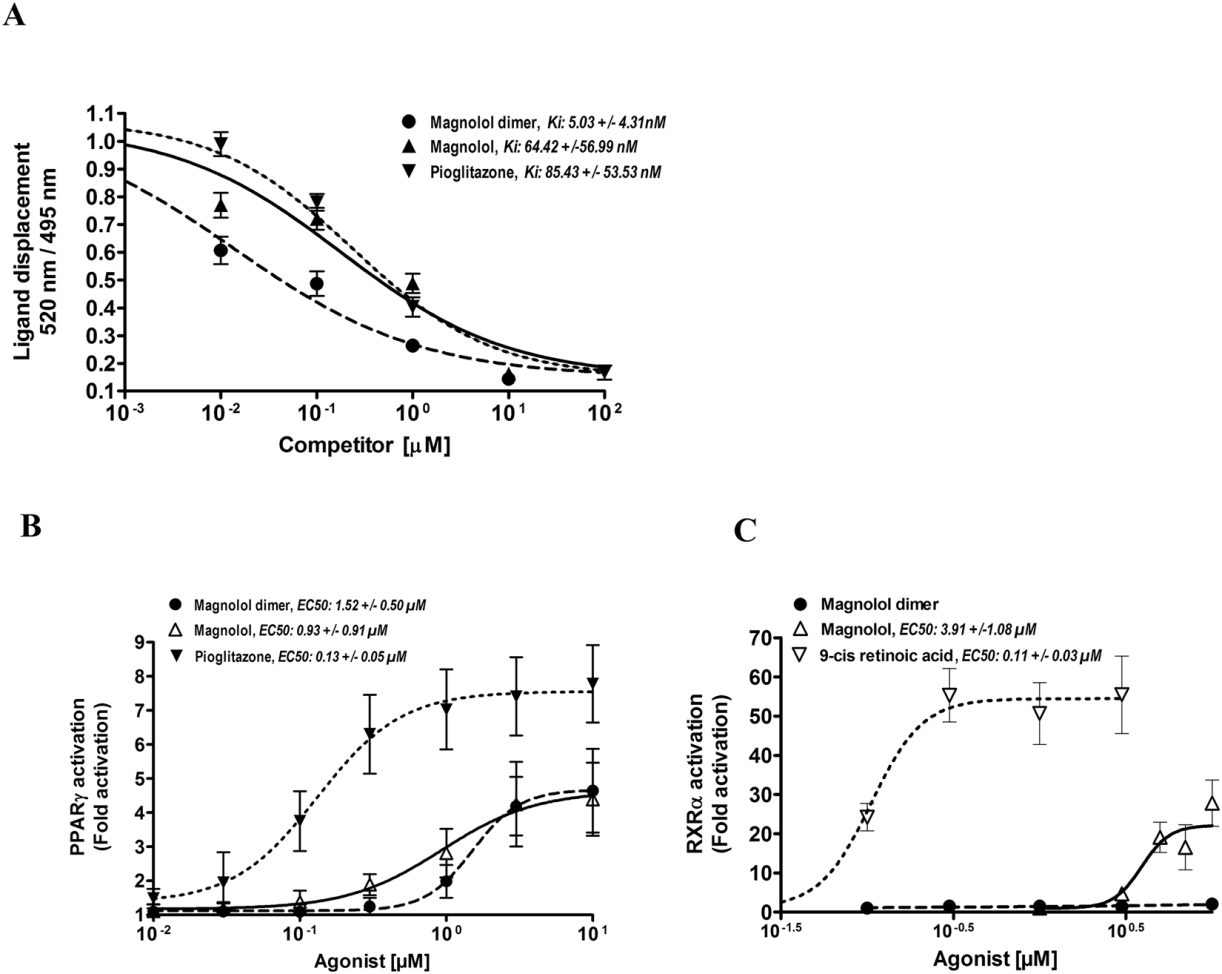
Binding of magnolol dimer (**1**), magnolol, and pioglitazone to the PPARγ ligand binding domain in a competitive *in vitro* assay (**A**). The different compounds were serially diluted in DMSO and incubated with purified human PPARγ LBD tagged with GST, a terbium-labeled anti-GST antibody, and fluorescently labeled PPARγ agonist (Fluormone™ Pan-PPAR Green). Compounds that bind to the PPARγ LBD displace the fluorescently labeled ligand leading to a fluorescence resonance energy transfer (FRET). Binding is estimated from the decrease of the emission ratio 520 nm/495 nm upon excitation at 340 nm. Data points are represented as mean ± SEM from four independent experiments performed in duplicate. *K*_i_ values were calculated using a sigmoidal curve fit with variable slope and 95% confidence intervals are shown. Nuclear receptor-dependent luciferase reporter transactivation of PPARγ (**B**) and RXRα (**C**) by magnolol dimer (**1**) in comparison to magnolol. (**B**) PPARγ-mediated luciferase reporter gene transactivation by magnolol dimer (**1**), magnolol and pioglitazone. HEK-293 cells were transfected as described in the Methods section and stimulated for 18 h, as indicated. Luciferase activity was normalized to EGFP-derived fluorescence. Results are expressed as fold induction compared with the solvent control (DMSO, 0.1%). EC_50_ values were calculated using a sigmoidal curve fit with variable slope. (**C**) RXRα-mediated luciferase reporter gene transactivation by magnolol and the full RXR agonist 9-cis retinoic acid. HEK293 cells were transfected as described in the Methods section, treated as indicated for 18 h and luciferase activity measured as under (**A**). All data are shown as means ± SD of at least three independent experiments performed in quadruplicate. EC50 values were calculated and values are indicated together with 95% confidence intervals.

To evaluate the binding of magnolol dimer (**1**) to the nuclear receptor PPARγ, we performed a binding assay with the purified human PPARγ LBD (LanthaScreen® TR-FRET PPARγ competitive binding assay). In this assay a fluorescently labeled ligand (Fluormone, Invitrogen) is displaced upon binding of the tested compound leading to a decrease in the FRET signal. Binding to the receptor was assessed in a concentration-dependent manner. Direct binding of magnolol dimer (**1**) to the PPARγ LBD *in vitro* was compared to the binding of magnolol and the full agonist pioglitazone (positive control). We found that magnolol dimer (**1**) binds with a more than 12-fold higher affinity to human PPARγ LBD compared to magnolol (*K*_i_ values of 5.03 nM, 95% CI 2.31-10.93 nM, and 64.42 nM, 95% CI 29.02-143.00 nM, respectively) and with more than 16-fold higher affinity compared to pioglitazone (*K*_i_ values of 5.03 nM, 95% CI 2.31-10.93 nM and 85.43, 95% CI 47.28-154.30 nM, respectively) (Fig. 7A).

Prior to cellular assays, we assessed possible cytotoxic effects of magnolol dimer (**1**) employing a resazurin conversion assay in HEK293 cells (Supplementary Information). Magnolol dimer (**1**) as well as magnolol did not reduce cell viability below 90% at 10 µM, however higher concentrations of magnolol dimer (**1**) and magnolol (30 µM) strongly reduced cell viability in this assay system (22.67 ± 5.35% cell viability at 30 µM of magnolol dimer and 9.1 ± 9.05% cell viability at 30 µM of magnolol, respectively) (Supplementary Information, Fig. 1). Based on these data, we decided to perform pharmacological dose-response experiments in cells only up to 10 µM.

We then assessed whether magnolol dimer (**1**) is a functional agonist and is able to promote PPARγ-dependent luciferase gene expression. To exclude RXRα activity, we also evaluated RXRα-dependent luciferase gene expression. HEK293 cells were transfected with an expression plasmid encoding for the full-length nuclear receptors PPARγ or RXRα and a reporter plasmid encoding the respective nuclear receptor response element coupled to a luciferase gene. To account for differences in cell number an EGFP plasmid was co-transfected as internal control. We compared the ability to concentration-dependently activate PPARγ or RXRα of magnolol dimer (**1**), magnolol, and the respective positive controls (pioglitazone for PPARγ and 9-cis retinoic acid for RXRα) by incubating the transfected cells with the compounds for 18 hours.

We found that magnolol dimer (**1**) promotes PPARγ-dependent luciferase gene expression comparably effective as magnolol (EC_50_ values of 1.52 µM, 95% CI 1.10-2.10 µM and 0.93 µM, 95% CI 0.39-2.22 µM, respectively). In contrast to magnolol the dimer did not transactivate RXRα-dependent luciferase gene expression showing that it possessed a higher specificity for PPARγ compared to magnolol (Fig. 7B,C).

**Figure 8:**
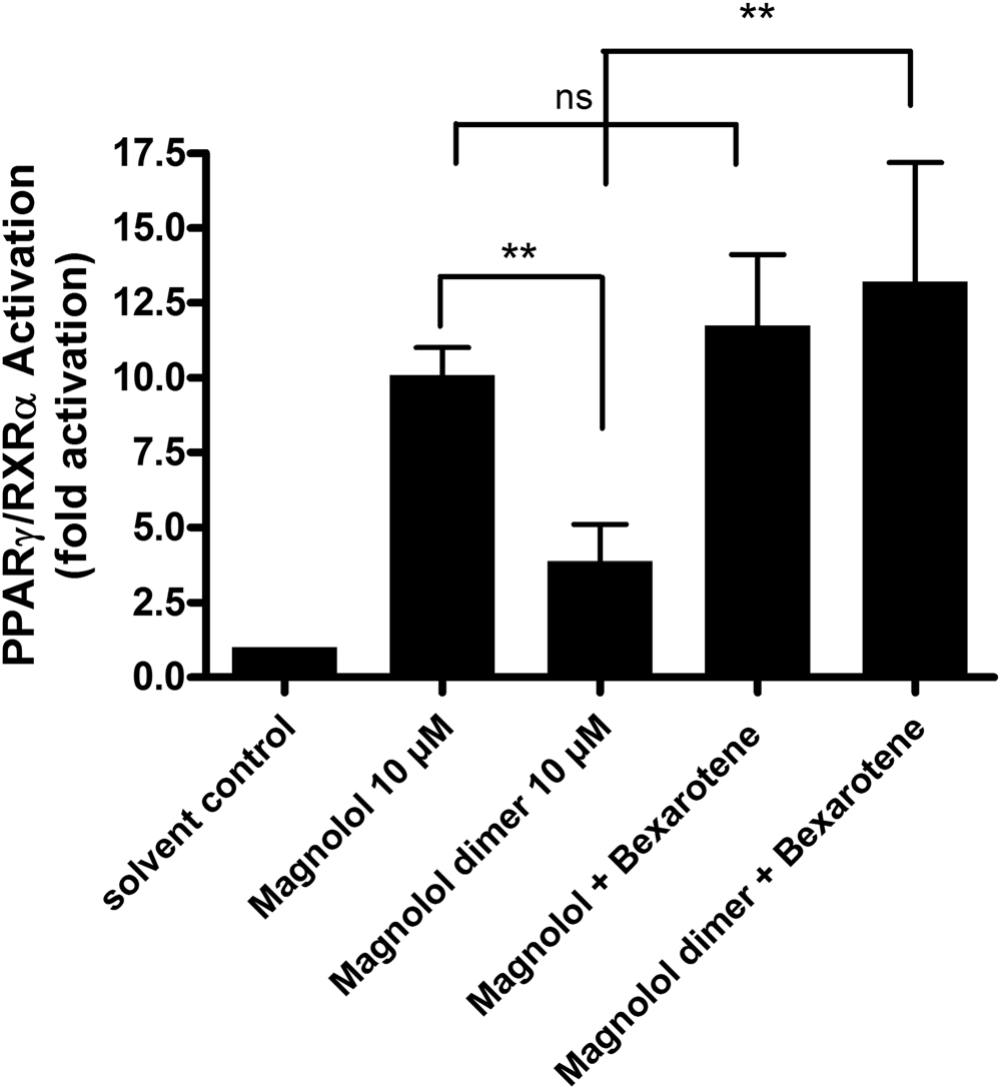
PPARγ/RXRα heterodimer-induced luciferase reporter transactivation upon treatment with magnolol, magnolol dimer (**1**), bexarotene (a full RXRα agonist) and co-treatments with these ligands. HEK-293 cells were transfected with expression vectors for PPARγ and RXRα, as described in the Methods section, and were stimulated for 18 h, as indicated. Luciferase activity was normalized to EGFP-derived fluorescence. Results are expressed as fold induction compared with the solvent control (DMSO, 0.2%). Data are expressed as means ± SD of at least three independent experiments performed in quadruplicate. Statistical significance was calculated by using One-way ANOVA and a Bonferroni post-test (p < 0.05*, p < 0.01**, ns not significant).

Furthermore, we compared the transactivation capacity of magnolol and magnolol dimer (**1**) at saturating concentrations (10 µM) on the PPARγ/RXRα heterodimer by co-transfecting HEK293 cells with both nuclear receptors and a PPAR response element coupled to a luciferase reporter gene. Under these conditions magnolol activates the heterodimer 10.08 ± 0.92 fold whereas the magnolol dimer (**1**) displays 3.82 ± 1.22 fold activation, a comparable activity as in a single transfection experiment with PPARγ. The higher activity of magnolol under these experimental conditions is in line with the dimer’s selectivity for PPARγ, and magnolol’s ability to bind to both PPARγ and RXRα^10^. Furthermore, in line with fully occupied PPARγ and RXRα LBDs in response to magnolol, co-treatment with the full RXR agonist bexarotene and magnolol revealed no significant additive effect (10.08 ± 0.92 fold activity *versus* 11.72 ± 2.38) whereas this was the case for magnolol dimer (**1**)(3.82 ± 1.22 fold activity *versus* 13.20 ± 3.98) binding only to PPARγ (Fig. 8).

PPARγ plays a major role in adipocyte differentiation and in the regulation of adipose-related genes that are involved in developing the adipose phenotype^25^. To verify that the magnolol dimer (**1**) indeed acts as PPARγ agonist in a cellular system that endogenously expresses PPARγ, we determined the adipogenic potential of magnolol dimer (**1**) on 3T3-L1 preadipocytes. Indeed magnolol dimer (**1**) concentration-dependently (1–10 µM) induces adipocyte differentiation (Supplementary Information, Fig. 2) similarly as shown for magnolol previously^8^.

## Discussion

Exploring new PPARγ ligands with improved and specific properties, we show here the computational design and the synthesis of a linked magnolol dimer based on the natural product magnolol. The ligand binding domain of PPARγ comprises a large Y-shaped ligand binding pocket that can be sub-divided into two pockets, one beta-sheet pocket and a pocket of the activation function 2 (AF-2) that lie in close proximity to each other. The crystal structure of the ligand binding domains of both PPARγ and RXRα that was previously published together with magnolol shows that two molecules of magnolol bind in each of the two PPARγ binding sub-pockets whereas the ligand binding domain of RXRα has only one binding pocket for magnolol^5,10^. Using this crystal structure, a magnolol dimer linked *via* an unsaturated chain was manually designed within the PPARγ binding site.

The herein reported synthesis of magnolol dimer (**1**) features an efficient, straightforward access to the target compound. Starting from a simple, commercial building block, the A/B ring fragment was obtained by a consecutive halogenation and coupling strategy. The challenging isomerization behavior of the allylic double bonds was successfully tackled by optimizing coupling conditions. The construction of the C/D ring fragment relied on a similar coupling methodology. With the two key intermediates - Wittig reagent **14** and aldehyde **8** - in hand, the C-C bond formation reaction furnished the olefination product very selectively in *Z*-configuration which was explicitly required by the structural guided design of magnolol dimer (**1**). Upon global demethylation the target molecule was obtained and provided sufficient material for the reported biological evaluation. The established route also enables preparation of simplified analogs which are being currently under investigation.

Our *in vitro* data show that the linked magnolol dimer has a much higher affinity to the ligand binding domain as magnolol and pioglitazone. The calculated *K*_i_ values in a FRET-based competitive binding assay are 5.03 ± 4.31 nM for magnolol dimer (**1**), 64.42 ± 56.99 nM for magnolol, and 85.43 ± 53.51 nM for pioglitazone. The much higher affinity of magnolol dimer (**1**) to PPARγ may be a result of a better binding of the dimer in the sub-pockets of the ligand binding domain as compared to two simultaneously binding magnolol molecules.

To verify functional activity of magnolol dimer (**1**) in a cellular system and to test its selectivity *versus* its heterodimerzation partner RXRα, we examined PPARγ- and RXRα-dependent gene transactivation in HEK293 cells. Magnolol dimer (**1**) activates PPARγ-dependent luciferase gene expression with about 1.5 fold lower potency compared to magnolol (EC_50_ values of 1.52 ± 0.50 µM and 0.93 ± 0.91 µM, respectively) but with comparable efficacy as magnolol, that is around 62% compared to pioglitazone (E_max_ values ± SD of magnolol dimer (**1**) 4.67 ± 0.26, magnolol 4.63 ± 0.55 and pioglitazone 7.56 ± 0.27) meaning that magnolol dimer (**1**) shows - as magnolol - partial agonism for PPARγ in our cellular system. In contrast to magnolol, magnolol dimer (**1**), however, does not activate RXRα-dependent luciferase gene expression proving selectivity of the designed linked dimer for PPARγ *versus* RXRα. Partial agonists, per definition, are able to bind to and activate a specific receptor, but show only partial efficacy at this receptor compared to a respective full agonist. Partial and less potent agonists of the nuclear receptor PPARγ have been proposed to produce fewer side effects as compared to full agonists and have been defined as selective PPARγ modulators^26^. Thus, magnolol dimer (**1**) can be considered as selective PPARγ modulator with specificity for PPARγ (versus RXRα).

Three magnolol molecules are binding to the PPARγ:RXRα heterodimer: two magnolol molecules to the PPARγ LBD and one molecule to the RXRα LBD^10^. In contrast, only one molecule of magnolol dimer (**1**) binds to the PPARγ LBD. Thus, it appears at first sight surprising that the potency of magnolol is nevertheless slightly higher than that of magnolol dimer (**1**). A possible explanation might be the additive activation of both, PPARγ and RXRα, in the case of magnolol that is lacking in the case of magnolol dimer (**1**), since it does not induce RXRα-mediated transcriptional activity. This mechanism of action is supported by the results of the PPARγ/ RXRα co-transfection experiments in Fig. 8. In this setting, the magnolol dimer (**1**) acts as even more partial agonist in comparison to magnolol and co-treatment with the RXR agonist bexarotene results in an additive effect.

Overall, magnolol dimer (**1**) shows a high affinity for the nuclear receptor PPARγ and is selective (versus RXRα) for this nuclear receptor, whereas magnolol possesses dual activity on the PPARγ:RXRα heterodimer. Thus, with magnolol and the newly developed dimer at hand it would appear feasible to differentiate between genes favorably induced by PPARγ/RXRα heterodimers with only PPARγ activated (magnolol dimer (**1**)) and both heterodimer partners activated (magnolol), respectively. Consequently, the rationally designed construct may represent a valuable pharmacological tool for further assessment. Moreover, the study shows that the combination of structural biology and *in silico* approaches can guide the smart chemical synthesis allowing for a better understanding, controlling and overcoming polypharmacology of natural products when needed.

## Methods

### Chemicals, Cell Culture Reagents, and Plasmids

LanthaScreen® TR-FRET PPARγ Competitive Binding Assay Kit was purchased from Invitrogen (Lofer, Austria). Pioglitazone was purchased from Molekula Ltd (Shaftesbury, UK) and 9-cis retinoic acid from Cayman Chemical (Michigan, USA). Magnolol was isolated from *Magnolia officinalis* as previously described^8^. Dulbecco’s modified Eagle’s medium (DMEM) containing 4.5 g/liter glucose was purchased from Lonza Group AG (Basel, Switzerland) and fetal bovine serum from Invitrogen (Lofer, Austria). The PPAR luciferase reporter construct (tk-PPREx3-luc) was a kind gift from Prof. Ronald M. Evans (Howard Hughes Medical Institute, La Jolla, CA). The expression plasmid for human PPARγ1 (pSG5-PL-PPARγ) was a kind gift of Prof. Walter Wahli and Prof. Beatrice Desvergne (Center for Integrative Genomics, University of Lausanne, Switzerland).The RXRα luciferase reporter vector was from Panomics Affymetrix (Milano, Italia) and the expression plasmid for human RXRα was purchased from Missouri S&T (Missouri, USA). DMSO was used as solvent and solvent vehicle controls were always included. For cell-based assays final concentrations never exceed 0.1% DMSO.

### Computational design of the magnolol dimers

For the computational design of the magnolol dimer, LigandScout 3.1 was employed^27^. The linker chains were grown by replacing a hydrogen on the initial magnolol by a carbon. From this new carbon, another hydrogen was replaced so that the linker pointed into the direction of the other bound magnolol molecule. This way, the linkers were designed step-by-step directly within the 3D ligand binding domain of PPARγ. Energy minimization of the designed dimers and the surrounding binding site amino acids was performed using the MMFF94 force field as implemented in LigandScout.

### Nuclear receptor luciferase reporter gene transactivation

Transactivation experiments were performed in HEK293 cells (ATCC, Manassas, VA) that were maintained in DMEM supplemented with 2 mM glutamine, 100 U/ml benzylpenicillin, 100 μg/ml streptomycin, and 10% fetal bovine serum. 6 × 10^6^ cells were seeded in 20 cm dishes, cultured for 18 h and then transfected with 6 μg of full-length human PPARγ and full-length human RXRα expression plasmid, respectively or both, 6 μg of the respective firefly luciferase reporter, and 3 μg of pEGFP-N1 (Clontech, CA, USA) as internal control. Six hours later, cells were reseeded in 96-well plates (4 × 10^4^ cells/well) in serum-free DMEM supplemented with 2 mM glutamine. Reseeded cells were treated as indicated and incubated for 18 h. After cell lysis luciferase activity and EGFP fluorescence were quantified on a GeniosPro plate reader (Tecan, Grödig, Austria). The ratio of luminescence units to fluorescence units was calculated to account for differences in cell number or transfection efficiency. Results were expressed as fold induction compared to the solvent DMSO (0.1%).

### PPARγ competitive ligand binding

Binding of magnolol dimer (**1**), magnolol and pioglitazone to the PPARγ ligand binding domain (LBD) was assessed using a time-resolved fluorescence resonance energy transfer (TR-FRET) PPARγ competitive binding assay (LanthaScreen®, Invitrogen, Lofer, Austria) according to the manufacturer’s protocol. The compounds were dissolved in DMSO (solvent vehicle) and incubated together with the human PPARγ LBD tagged with GST, terbium-labeled anti-GST antibody and fluorescently labeled PPAR ligand (Fluormone™ Pan-PPAR Green). Binding of a ligand results in a fluorescence resonance energy transfer (FRET) after excitation of terbium at 340 nm and in an emission at 520 nm. Test compounds binding to the human PPARγ LBD are competing with the fluorescently labeled ligand and displacing it, resulting in a decrease of the FRET signal. The signal obtained at 520 nm is normalized to the signal obtained from the terbium emission at 495 nm. The decrease in the 520 nm/495 nm ratio is used as a measure for the ability of the tested compounds to bind to the human PPARγ LBD. Neither pioglitazone nor honokiol interfered with the background 520 nm/495 nm fluorescence in the absence of PPARγ LBD. All quantifications were performed with a GeniosPro plate reader (Tecan).

### Statistical Methods and Data Analysis

Statistical analysis and nonlinear regression were performed using Prism software (ver. 4.03; GraphPad Software Inc., San Diego, CA). To calculate the EC_50_ values data were curve fitted and non-linear transformed using a sigmoidal dose response with variable slope. *K*_i_ values of competitor compounds were calculated with the Cheng-Prusoff equation: (*K*_i_) = IC_50_/(1 + *L*/*K*_D_)^28^.

## Electronic supplementary material

Supplemental Material
