# Supplementary material for: Linked magnolol dimer as a selective PPARγ agonist – Structure-based rational design, synthesis, and bioactivity evaluation
Source: Sci Rep. 2017 Oct 20;7:13002. doi: 10.1038/s41598-017-12628-5 (PMC5651862; doi:10.1038/s41598-017-12628-5)
Supplement: Supplementary file 1 — Supplemental Material [file 41598_2017_12628_MOESM1_ESM.pdf]

# Supplementary Information

## Linked magnolol dimer as a selective PPARγ agonist – Structure-based rational design, synthesis, and bioactivity evaluation

Dominik Dreier[1,#], Simone Latkolik[2,#], Lukas Rycek[1], Michael Schnürch[1], Andrea Dymáková[2,5], Atanas G. Atanasov[2,6], Angela Ladurner[2], Elke H. Heiss[2], Hermann Stuppner[3], Daniela Schuster[4], Marko D. Mihovilovic[1] & Verena M. Dirsch[2]

[1]Institute of Applied Synthetic Chemistry, TU Wien, Vienna, Austria. [2]Departmet of Pharmacognosy, University of Vienna, Vienna, Austria. [3]Institute of Pharmacy/Pharmacognosy, Center for Chemistry and Biomedicine, University of Innsbruck, Innsbruck, Austria. [4]Institute of Pharmacy/Pharmaceutical Chemistry, Center for Chemistry and Biomedicine, University of Innsbruck, Innsbruck, Austria. [5]Department of Biochemical Sciences, Faculty of Pharmacy in Hradec Králové, Charles University, Hradec Králové, Czech Republic. [6]Institute of Genetics and Animal Breeding of the Polish Academy of Sciences, 05-552 Jastrzebiec, Poland. [#]These authors contributed equally to this work. Correspondence and requests for materials should be addressed to Verena.Dirsch@univie.ac.at or Daniela.Schuster@uibk.ac.at

## General Notes

Microwave reactions were carried out in a BIOTAGE® Initiator sixty. GC/MS spectra were measured on a Thermo Finnigan system: GC: Focus GC with a BGB5 column (l = 30 m, di = 0.25 mm, 0.25 µm film), MS: DSQ II with quadrupol (EI). For thin layer chromatography aluminium backed silica gel 60 F254 (Merck) was used. Medium pressure liquid chromatography was performed on a Büchi SepacoreTM Flash System. Pump-System: 2x Büchi Pump Module C-605, Büchi Pump Manager C-615; detector: Büchi UV Photometer C-635; fraction collector: Büchi Fraction Collector C-660. Carrier material was silica gel 60 (Merck, 40-63 µm). $^{1}$H-NMR and $^{13}$C-NMR spectra were recorded from $CDCl_3$ solutions on a Bruker AC 200 (200 MHz), a Bruker Avance UltraShield 400 (400 MHz) or a Bruker Avance III HD 600 (600 MHz) spectrometer. Chemical shifts are reported in ppm relative to the nominal residual solvent signal of $CDCl_3$: $^{1}$H: 7.26 ppm, $^{13}$C: 77.0 ppm Melting points were recorded on a Büchi B-545 melting point apparatus. HRMS were measured at Shimadzu HPLC-IT-TOF mass spectrometer with either APCI or ESI ionization method.

# Synthesis

2-(4-Methoxyphenyl)acetaldehyde **4**

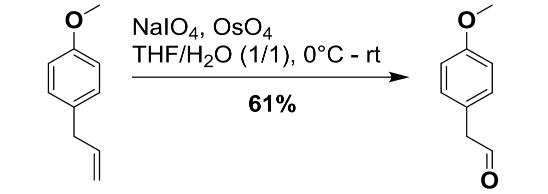


(**3**) 148.21 g/mol → **4** 150.18 g/mol

| | |
|---|---|
| **Procedure** | $NaIO_4$ (4280 mg, 20.01 mmol, 3 equiv.) and $OsO_4$ (17 mg, 0.01 mmol, 1mol%) were added to an ice bath cooled solution of freshly distilled 1-allyl-4-methoxybenzene (**3**) (1000 mg, 6.75 mmol, 1 equiv.) in degassed THF/$H_2O$ (40 mL, 1/1) under argon. The ice bath was removed and the solution was stirred at room temperature until TLC indicated complete consumption of the starting material (25 minutes). The reaction mixture was quenched with satd. aqu. $Na_2S_2O_3$ (40 mL), extracted with diethyl ether (2 x 40 mL) and the combined organic layers were dried over $MgSO_4$. The solvent was removed in *vacuo* and after purification by *Kugelrohr* distillation in high vacuum the product was obtained. |
| **Yield** | 61% (618 mg, 4.115 mmol) |
| **Appearance** | colorless oil |
| **Sum formula** | $C_9H_{10}O_2$ |
| **TLC** | $R_f$ (LP/EtOAc = 5/1) = 0.5 |
| **$^1$H-NMR (200 MHz, $CDCl_3$)** | $\delta$ = 3.63 (d, *J* = 2.4 Hz, 2H), 3.81 (s, 3H), 6.90 (d, *J* = 8.7 Hz, 2H), 7.13 (d, *J* = 8.6 Hz, 2H), 9.72 (t, *J* = 2.4 Hz, 1H) ppm. |
| **$^{13}$C-NMR (50 MHz, $CDCl_3$)** | $\delta$ = 49.8, 55.4, 114.6 (2C), 123.8, 130.8 (2C), 159.1, 199.8 ppm. |
| **GC-MS** | 150 (17, $M^+$), 122 (10), 121 (100) |
| **Comment** | Spectral data are in accordance with literature reports.[1] |

2-(4-Methoxybenzyl)-1,3-dioxolane **5**

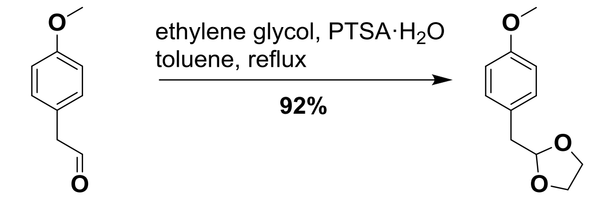


| | |
|---|---|
| **Procedure** | A 100 mL flask was charged with aldehyde **4** (3.54 g, 23.6 mmol, 1 equiv) and *p*-toluenesulfonic acid monohydrate (134 mg, 0.71 mmol, 3 mol%) under argon, then dry toluene (34 mL) and ethylene glycol (5.27 g, 84.9 mmol, 3.6 equiv) were added. The reaction solution was stirred under reflux on a Dean-Stark apparatus for 24 hours. Then the reaction solution was cooled to room temperature and was washed with satd. aqu. $NaHCO_3$ (40 mL) and brine (3 x 40 mL) before being dried over $MgSO_4$. The mixture was filtered and evaporation of the solvent in *vacuo* provided the desired product. |
| **Yield** | 92% (4.231 g, 21.8 mmol) |
| **Appearance** | orange liquid |
| **Sum formula** | $C_{11}H_{14}O_3$ |
| **TLC** | $R_f$ (LP/EtOAc = 5/1) = 0.5 |
| **$^1$H-NMR (200 MHz, $CDCl_3$)** | $\delta$ = 2.91 (d, *J* = 4.8 Hz, 2H), 3.79 (s, 3H), 3.81 – 3.99 (m, 4H), 5.02 (t, *J* = 4.8 Hz, 1H), 6.85 (d, *J* = 8.7 Hz, 2H), 7.19 (d, *J* = 8.6 Hz, 2H) ppm. |
| **$^{13}$C-NMR (50 MHz, $CDCl_3$)** | δ = 39.9, 55.3, 65.0 (2C), 104.9, 113.9 (2C), 128.2, 130.7 (2C), 158.4 ppm. |
| **GC-MS** | 194 (9, $M^+$), 121 (32), 73 (100) |
| **Comment** | Spectral data are in accordance with literature reports.[2] |

2-(3-Bromo-4-methoxybenzyl)-1,3-dioxolane **6**

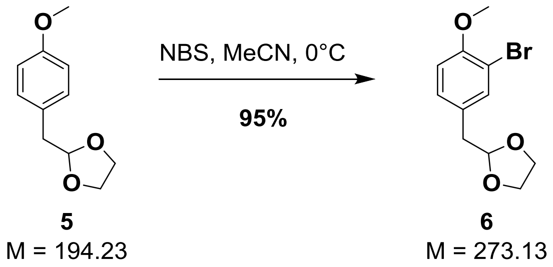


| | |
|---|---|
| **Procedure** | A 100 mL flask was charged with acetale **5** (1000 mg, 5.15 mmol, 1 equiv.) and MeCN (30 mL) and the reaction solution was cooled to 0 °C. NBS (916 mg, 5.15 mmol, 1 equiv.) was added and the reaction solution was stirred at 0°C for 2 hours. The solvent was removed in *vacuo*. The residue was distributed between diethyl ether (40 mL) and $H_2O$ (40 mL) and the two phases were separated. The aqu. phase was extracted with diethyl ether (2 x 15 mL) and the combined organic layers were dried over $MgSO_4$. After the solvent was removed in *vacuo* the product was obtained. |
| **Yield** | 95% (1336 mg, 4.89 mmol) |
| **Appearance** | dark-orange oil |
| **Sum formula** | $C_{11}H_{13}BrO_3$ |
| **TLC** | $R_f$ (LP/EtOAc = 5/1) = 0.35 |
| **$^1$H-NMR (200 MHz, $CDCl_3$)** | $\delta$ = 2.88 (d, *J* = 4.7 Hz, 2H), 3.82 – 3.95 (m, 4H), 3.87 (s, 3H), 5.02 (t, *J* = 4.7 Hz, 1H), 6.83 (d, *J* = 8.4 Hz, 1H), 7.17 (dd, *J* = 8.4 Hz & 2.1 Hz, 1H), 7.47 (d, *J* = 2.1 Hz, 1H) ppm. |
| **$^{13}$C-NMR (50 MHz, $CDCl_3$)** | $\delta$ = 39.4, 56.2, 65.0 (2C), 104.3, 111.4, 111.8, 129.7, 129.8, 134.4, 154.6 ppm. |
| **GC-MS** | 274 (2, $M^+$), 272 (2, $M^+$), 73 (100) |

2-(5-Allyl-2-methoxyphenyl)-4,4,5,5-tetramethyl-1,3,2-dioxaborolane **7**

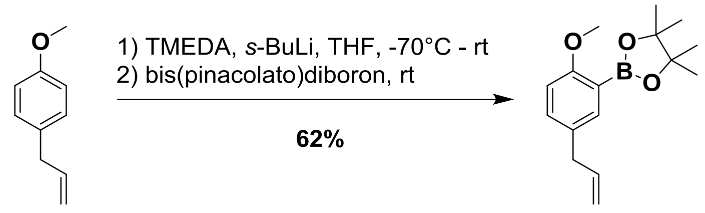


(**3**)
M = 148.20

**7**
M = 274.17

| | |
|---|---|
| **Procedure** | Freshly distilled 1-allyl-4-methoxybenzene (**3**) (1000 mg, 6.75 mmol, 1 equiv.) was charged into an oven dried 50 mL flask. The flask was closed with a septum and was evacuated and flushed with argon on a Schlenk line three times. Dry THF (25 mL) was added *via* syringe and the solution was cooled to -70°C. Dry TMEDA (1.01 mL, 6.75 mmol, 1 equiv.) was added *via* syringe. *s*-BuLi (9.55 mL, 10.12 mmol, 1.5 equiv, 1.35M in cyclohexane, reagent was titrated prior to use according to a literature protocol[3]) was added dropwise *via* syringe over 3 minutes while a temperature of -75°C - -70°C was maintained. The reaction solution was stirred for 1 hour while the temperature was maintained between -70°C and -75°C. Then the reaction solution was allowed to reach room temperature within 10 - 15 minutes and was stirred for 30 minutes at room temperature. A solution of bis(pinacolato)diboron (1713 mg, 6.75 mmol, 1 equiv.) in dry THF (5 mL) was added dropwise *via* syringe. The reaction solution was stirred at room temperature for 1 hour. EtOAc (130 mL) was added and the reaction solution was treated with satd. aqu. $NH_4Cl$ (85 mL). The two phases were separated and the aqu. phase was extracted with EtOAc (2 x 20 mL). The combined organic layers were washed with satd. aqu. $NaHCO_3$ (100 mL) and brine (100 mL) to be finally dried over $MgSO_4$. The mixture was filtered and after evaporation of the solvent in *vacuo* the crude product was obtained. Subsequent purification by distillation in high vacuum provided the pure compound. |
| **Yield** | 62% (1148 mg, 4.18 mmol) |
| **Appearance** | pale yellow oil |
| **Sum formula** | $C_{16}H_{23}BO_3$ |
| **TLC** | $R_f$ (LP/EtOAc = 5/1) = 0.55 |
| **$^1$H-NMR (200 MHz, $CDCl_3$)** | *δ* = 1.36 (s, 12H), 3.33 (d, *J* = 6.7 Hz, 2H), 3.81 (s, 3H), 4.99 – 5.13 (m, 2H), 5.96 (ddt, *J* = 16.9 Hz & 10.2 Hz & 6.6 Hz, 1H), 6.80 (d, *J* = 8.5 Hz, 1H), 7.21 (dd, *J* = 8.4 Hz & 2.4 Hz, 1H), 7.49 (d, *J* = 2.4 Hz, 1H) ppm. |
| **$^{13}$C-NMR (50 MHz, $CDCl_3$)** | *δ* = 24.9 (4C), 39.4, 56.1, 83.6 (2C), 110.8, 115.4, 131.5, 132.6, 136.8, 138.1, 162.8 ppm. C1 not detected |
| **GC-MS** | 274 (71, $M^+$), 273 (19), 201 (39), 175 (26), 174 (100), 173 (69), 159 (21), 158 (31), 157 (15), 147 (38), 146 (29), 145 (27), 143 (20), 131 (70), 129 (22), 117 (27), 116 (38), 115 (76) |

| | |
|---|---|
| **HRMS** | $[M+Na]^+$: calculated: 297.1635 Da, found: 297.1626 Da, difference: 0.9 mDa |

2-((5'-Allyl-2',6-dimethoxy-[1,1'-biphenyl]-3-yl)methyl)-1,3-dioxolane **8**

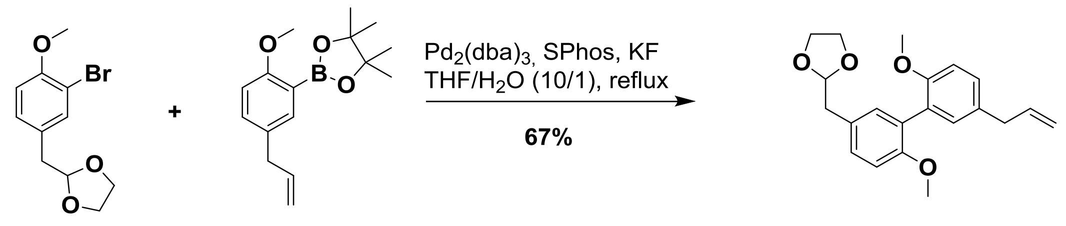


**6** M = 273.13 **7** M = 274.17 **8** M = 340.42

| | |
|---|---|
| **Procedure** | Bromide **6** (720 mg, 2.64 mmol, 1 equiv.), boronic ester **7** (795 mg, 2.90 mmol, 1.1 equiv.), $Pd_2(dba)_3$ (241 mg, 0.26 mmol, 10mol%), SPhos (325 mg, 0.79 mmol, 30mol%), KF (766 mg, 13.18 mmol, 5 equiv.), THF (24 mL), and $H_2O$ (2.4 mL) were charged into a 50 mL flask. The flask was closed with a septum and was evacuated and flushed with argon on a Schlenk line three times. The flask was attached to a reflux condenser and was stirred under reflux for 24 hours. The reaction solution was cooled to room temperature and was diluted with EtOAc (75 mL). The reaction solution was washed with satd. aqu. $NH_4Cl$ (50 mL). The aqu. phase was extracted with EtOAc (2 x 15 mL). The combined organic layers were washed with $NaHCO_3$ (50 mL) and brine (50 mL). The organic phase was dried over $MgSO_4$. After evaporation of the solvent in *vacuo* and purification by flash column chromatography (crude mass/$SiO_2$ = 1/55, 5 → 15% EtOAc in LP) the desired product was obtained. |
| **Yield** | 67% (601 mg, 1.77 mmol) |
| **Appearance** | colorless oil |
| **Sum formula** | $C_{21}H_{24}O_4$ |
| **TLC** | $R_f$ (LP/EtOAc = 5/1) = 0.3 |
| **$^1$H-NMR (200 MHz, $CDCl_3$)** | $\delta$ = 2.96 (d, $J$ = 4.9 Hz, 2H), 3.39 (d, $J$ = 6.8 Hz, 2H), 3.77 (s, 6H), 3.81 – 4.03 (m, 4H), 5.02 – 5.19 (m, 3H), 6.01 (ddt, $J$ = 16.8 Hz & 10.0 Hz & 6.7 Hz, 1H), 6.92 (dd, $J$ = 8.3 Hz & 2.0 Hz, 2H), 7.07 – 7.19 (m, 3H), 7.25 (dd, $J$ =8.2 Hz & 2.5 Hz, 1H) ppm. |
| **$^{13}$C-NMR (50 MHz, $CDCl_3$)** | $\delta$ = 39.5, 40.1, 55.9, 56.0, 65.0 (2C), 105.0, 111.2, 111.3, 115.6, 127.8, 127.9, 128.5, 129.7, 131.8, 131.8, 132.8, 137.9, 155.6, 156.0 ppm. 1 quaternary carbon (C3 or C5') superimposed or not detected |
| **GC-MS** | 340 (5, $M^+$), 73 (100) |
| **HRMS** | $[M+H]^+$: calculated: 341.1747 Da, found: 341.1759 Da, difference: 1.2 mDa |

2-(5'-Allyl-2',6-dimethoxy-[1,1'-biphenyl]-3-yl)acetaldehyde **9**

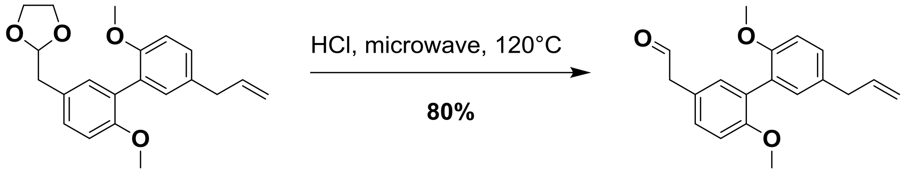


| | |
|---|---|
| **Procedure** | Acetale **8** (120 mg, 0.35 mmol) was placed in a 2 mL microwave vial. The vial was closed with a septum and evacuated and flushed with argon on a Schlenk line three times. 0.25 M degassed HCl (1.07 mL) was added by syringe. The reaction mixture was heated in the microwave oven (absorption level: high, 120°C) for 1 hour. The reaction mixture was extracted with EtOAc (3 x 1.5 mL). The combined organic layers were dried over $MgSO_4$. After evaporation of the solvent in *vacuo* the product was obtained. As the aldehyde could not be submitted to column chromatography due to pronounced decomposition and NMR-analysis indicated good purity, the material was used without purification for the next step. Purity was determined by $^1$H-NMR. The aldehyde signal and the signal from the adjacent methylene group were referenced to the signal from the allylic CH. A purity of 80% was determined while crude yield was quantitative. |
| **Yield** | approximately 80% (approximately 0.28 mmol) |
| **Appearance** | colorless oil |
| **Sum formula** | $C_{19}H_{20}O_3$ |
| **TLC** | $R_f$ (LP/EtOAc = 5/1) = 0.3 |
| **$^1$H-NMR (400 MHz, $CDCl_3$)** | $\delta$ = 3.40 (d, *J* = 6.7 Hz, 2H), 3.66 (d, *J* = 2.5 Hz, 2H), 3.78 (s, 3H), 3.80 (s, 3H), 5.06 – 5.16 (m, 2H), 6.01 (ddt, *J* = 16.8 Hz & 9.9 Hz & 6.7 Hz, 1H), 6.93 (d, *J* = 8.4 Hz, 1H), 6.99 (d, *J* = 8.4 Hz, 1H), 7.09 (d, *J* = 2.4 Hz, 1H), 7.12 (d, *J* = 2.3 Hz, 1H), 7.16 – 7.21 (m, 2H), 9.78 (t, *J* = 2.5 Hz, 1H) ppm. |
| **$^{13}$C-NMR (50 MHz, $CDCl_3$)** | $\delta$ = 39.5, 49.8, 55.9, 55.9, 111.2, 111.7, 115.7, 123.5, 127.3, 128.7, 128.8, 129.7, 131.6, 131.9, 132.7, 137.8, 155.5, 156.6, 200.0 ppm. |
| **GC-MS** | 296 (48, $M^+$), 268 (19), 267 (100), 221 (10), 211 (17), 194 (10), 165 (16), 152 (11), 133 (23) |
| **HRMS** | $[M+H]^+$: calculated: 297.1485 Da, found: 297.1489 Da, difference: 0.4 mDa |

1-(4-Chlorobutyl)-3-methoxybenzene **10**

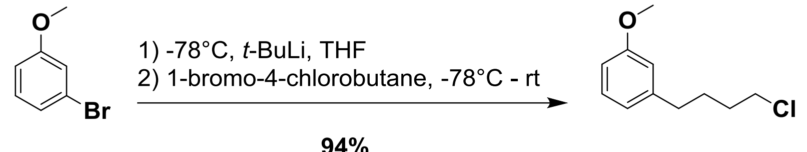


(**2**)
M = 187.04

**10**
M = 198.69

| | |
|---|---|
| **Procedure** | 1-Bromo-3-methoxybenzene (**2**) (3610 mg, 19.30 mmol, 1 equiv.) was placed in a 100 mL flask and dissolved in dry THF (38.6 mL). The flask was closed with a septum and was evacuated and flushed with argon on a Schlenk line three times. The reaction solution was cooled to -78 °C. A solution of *t*-BuLi (22.7 mL, 38.60 mmol, 2 equiv., 1.7 M in pentane) was added dropwise via syringe and the reaction solution was stirred for 15 minutes at -78 °C, then 10 minutes at -50 °C. The mixture was again cooled to -78°C and 1-bromo-4-chlorobutane (4964 mg, 28.95 mmol, 1.5 equiv.) was added whereupon the mixture was warmed to room temperature to be stirred subsequently for 1 hour. The reaction solution was then partitioned between satd. aqu. $NH_4Cl$ (90 mL) and DCM (90 mL). The aqu. layer was extracted with DCM (2 x 30 mL) and the combined organic layers were dried over $MgSO_4$. After evaporation of the solvent in vacuo a colorless liquid was obtained. The crude material was purified by flash column chromatography (crude mass/SiO2 = 1/50, 1% - 4% EtOAc in LP). After evaporation of the solvent in vacuo the product was obtained. |
| **Yield** | 94% (3586 mg, 18.05 mmol) |
| **Appearance** | colorless oil |
| **Sum formula** | $C_{11}H_{15}ClO$ |
| **TLC** | $R_f$ (LP/EtOAc = 5/1) = 0.8 |
| **$^1$H-NMR (200 MHz, $CDCl_3$)** | $\delta$ = 1.75 – 1.87 (m, 4H), 2.64 (t, *J* = 7.0 Hz, 2H), 3.56 (t, *J* = 6.3 Hz, 2H), 3.81 (s, 3H), 6.72 – 6.83 (m, 3H), 7.22 (dd, *J* = 8.9 Hz & 7.5 Hz, 1H) ppm. |
| **$^{13}$C-NMR (50 MHz, $CDCl_3$)** | $\delta$ = 28.5, 32.2, 35.2, 45.0, 55.2, 111.2, 114.3, 120.9, 129.4, 143.6, 159.8 ppm. |
| **GC-MS** | 200 (7, $M^+$), 198 (22, $M^+$), 163 (19), 122 (53), 121 (100) |

2-(4-Chlorobutyl)-1-iodo-4-methoxybenzene **11**

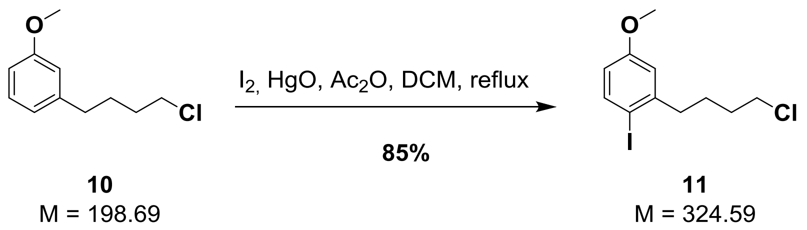


| | |
|---|---|
| **Procedure** | Chloride **10** (3239 mg, 16.30 mmol, 1 equiv.), HgO (3530 mg, 16.30 mmol, 1 equiv.), $Ac_2O$ (0.62 mL, 6.52 mmol, 0.4 equiv.), $I_2$ (8275 mg, 32.60 mmol, 1.4 equiv.) and DCM (60 mL) were placed in a 100 mL flask. The flask was flushed with argon and the mixture was stirred at room temperature for 22 hours. The reaction mixture was filtered through a pad of Celite® and the pad was washed with DCM and satd. aqu. $Na_2S_2O_3$. Phases were separated and the aqu. phase was extracted with DCM (3 x 10 mL). The combined organic layers were washed with brine (50 mL) and dried over $MgSO_4$. After evaporation of the solvent in *vacuo* and purification by flash column chromatography (crude mass/$SiO_2$ = 1/120, 3% EtOAc in PE) the product was obtained. |
| **Yield** | 85% (4498 mg, 13.86 mmol) |
| **Appearance** | colorless liquid |
| **Sum formula** | $C_{11}H_{14}ClIO$ |
| **TLC** | $R_f$ (5% EtOAc in PE) = 0.55 |
| **$^1$H-NMR (200 MHz, $CDCl_3$)** | $\delta$ = 1.65 – 1.96 (m, 4H), 2.70 (t, $J$ = 7.5 Hz, 2H), 3.58 (t, $J$ = 6.4 Hz, 2H), 3.78 (s, 3H), 6.51 (dd, $J$ = 8.7 Hz & 3.0 Hz, 1H), 6.78 (d, $J$ = 3.1 Hz, 1H), 7.67 (d, $J$ = 8.7 Hz, 1H) ppm. |
| **$^{13}$C-NMR (50 MHz, $CDCl_3$)** | $\delta$ = 27.6, 32.2, 40.2, 44.9, 55.5, 89.2, 113.8, 115.6, 140.0, 145.5, 160.1 ppm. |
| **GC-MS** | 326 (10, $M^+$), 324 (31, $M^+$), 247 (19), 162 (23), 161 (53), 122 (13), 121 (100), 120 (21) |

1-Bromo-4-(4-chlorobutyl)-5-iodo-2-methoxybenzene **12**

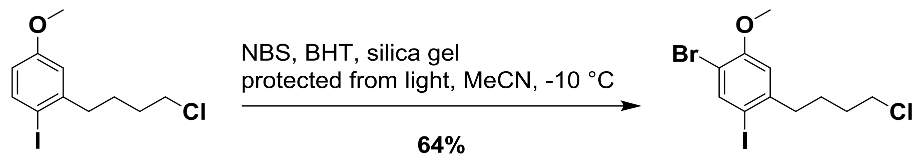


**11** M = 324.59 **12** M = 403.48

| | |
|---|---|
| **Procedure** | To a stirred solution of compound **11** (951 mg, 2.93 mmol, 1 equiv.) in MeCN (9.2 mL) in an aluminium foil coated flask was added silica gel (220 mg) and BHT (9.6 mg). The mixture was cooled to -10 °C and NBS (573 mg, 3.22 mmol, 1.1 equiv.) was added. The reaction was stirred at -10 °C for 48 hours. The mixture was filtered and the solids were wahed with DCM and the resulting solution was concentrated *in vacuo*. The residue was partitioned between DCM (20 mL) and water (20 mL). The aqu. phase was extracted with DCM (3 x 15 mL). The combined organic phase was dried over $MgSO_4$ and concentrated *in vacuo*. The obtained crude material was rotated on silica gel and was submitted to column chromatography (180 g silica gel, 3% EtOAc in PE). The obtained material was recrystallized from heptanes (approx. 1 mL). |
| **Yield** | 64% (753 mg, 1.87 mmol) |
| **Appearance** | colorless crystals |
| **Sum formula** | $C_{11}H_{13}BrClIO$ |
| **TLC** | $R_f$ (5% EtOAc in PE) = 0.5 |
| **$^1$H-NMR (200 MHz, $CDCl_3$)** | $\delta$ = 1.67 – 1.93 (m, 4H), 2.70 (t, $J$ = 7.6 Hz, 2H), 3.59 (t, $J$ = 6.3 Hz, 2H), 3.88 (s, 3H), 6.75 (s, 1H), 7.91 (s, 1H) ppm. |
| **$^{13}$C-NMR (50 MHz, $CDCl_3$)** | $\delta$ = 27.4, 32.0, 39.9, 44.7, 56.3, 88.7, 109.9, 112.6, 142.2, 145.0, 156.2 ppm. |
| **GC-MS** | 404 (51, $M^+$), 402 (40, $M^+$), 327 (44), 325 (45), 201 (93), 200 (31), 199 (100), 198 (24), 161 (74), 160 (34), 147 (42), 119 (18), 115 (30) |

1-Allyl-5-bromo-2-(4-chlorobutyl)-4-methoxybenzene **13**

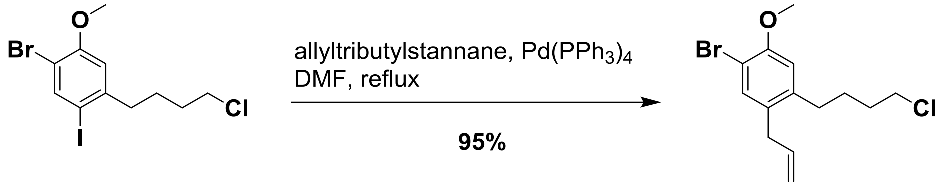


**12**
M = 403.48

**13**
M = 317.65

| | |
|---|---|
| **Procedure** | Compound **12** (1880 mg, 4.66 mmol, 1 equiv.), allyltributylstannane (1620 mg, 4.89 mmol, 1.05 equiv.), $Pd(PPh_3)_4$ (296 mg, 0.23 mmol, 5 mol%) and dry DMF (15.5 mL) were placed in an oven dried flask. The flask was flushed with argon and was attached to a reflux condenser. Reaction was heated with an oil bath (oil bath set to 110 °C) for 30 minutes. $H_2O$ (20 mL) was added and reaction was shaken with EtOAc (3 x 40 mL). The combined organic phase was washed with aqu. KF (10%, 60 mL), which caused precipitation. The mixture was filtered and residues were washed with EtOAc. Organic phase was shaken with aqu. KF (10%, 3 x 40 mL), washed with brine (25 mL) and subsequently dried over $MgSO_4$. After evaporation of the solvent *in vacuo* the crude product was obtained which was purified by flash column chromatography (180 g silica gel, 1% EtOAc in LP). |
| **Yield** | 95% (1406 mg, 4.43 mmol) |
| **Appearance** | pale yellow liquid |
| **Sum formula** | $C_{14}H_{18}BrClO$ |
| **TLC** | $R_f$ (5% EtOAc in PE) = 0.4 |
| **$^1$H-NMR (200 MHz, $CDCl_3$)** | $\delta$ = 1.63 – 1.94 (m, 4H), 2.59 (d, $J$ = 7.9 Hz, 2H), 3.30 (d, $J$ = 6.2 Hz, 2H), 3.57 (t, $J$ = 6.2 Hz, 2H), 3.88 (s, 3H), 4.91 – 5.12 (m, 2H), 5.91 (ddt, $J$ = 16.5 Hz & 10.1 Hz & 6.2 Hz, 1H), 6.69 (s, 1H), 7.30 (s, 1H) ppm. |
| **$^{13}$C-NMR (50 MHz, $CDCl_3$)** | $\delta$ = 28.1, 32.2, 32.4, 36.1, 44.9, 56.4, 108.9, 113.0, 116.1, 131.3, 134.3, 137.0, 140.7, 154.3 ppm. |
| **GC-MS** | 318 (33, $M^+$), 316 (25, $M^+$), 241 (23), 239 (29), 237 (19), 161 (18), 160 (80), 159 (36), 147 (27), 146 (100), 145 (40), 131 (14), 129 (24), 128 (22), 117 (17), 116 (15), 115 (44), 103 (11) |

5,5'-Diallyl-4-(4-chlorobutyl)-2,2'-dimethoxy-1,1'-biphenyl **14**

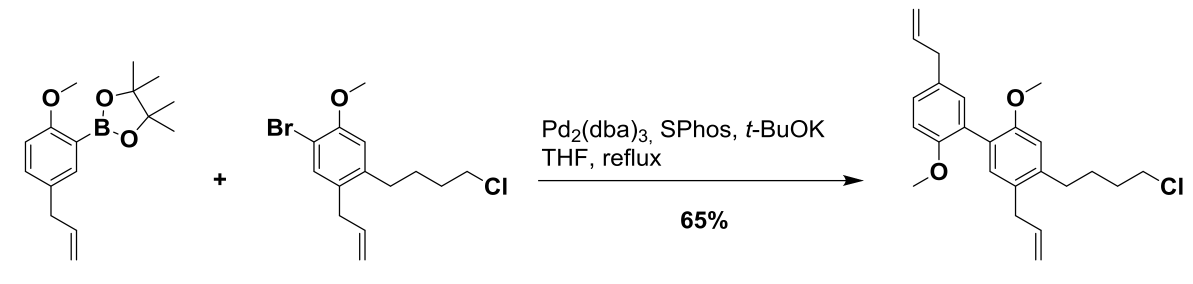


**7** M = 274.17 | **13** M = 317.65 | **14** M = 384.94

**Procedure** Bromide **13** (100 mg, 0.31 mmol, 1 equiv.), boronic ester **7** (95 mg, 0.35 mmol, 1.1 equiv.), $Pd_2(dba)_3$ (29 mg, 0.03 mmol, 10 mol%), S-Phos (39 mg, 0.09 mmol, 30 mol%) and *t*-BuOK (106 mg, 0.94 mmol, 3 equiv.) were weighted into a vial. THF (3.15 mL) was added. The vial was flushed with argon and the reaction was heated under reflux for 21 hours (thermoblock set to 72 °C). $H_2O$ (1.5 mL) was added and phases were separated. The aqu. phase was extracted with EtOAc (3 x 1.5 mL). The combined organic phase was washed with satd. aqu. $NaHCO_3$ (4 mL) and brine (4 mL) and was dried over $MgSO_4$. The mixture was filtered through a short pad of silica gel. After evaporation of the solvent the crude product was purified ba flash comlumn chromatography (18 g silica gel, 1% - 2% EtOAc in LP).

**Yield** 65 % (79 mg, 0.20 mmol)

**Appearance** pale yellow oil

**Sum formula** $C_{24}H_{29}ClO_2$

**TLC** $R_f$ (LP/EtOAc = 10/1) = 0.4

**$^1$H-NMR (200 MHz, $CDCl_3$)** $\delta$ = 1.73 – 2.03 (m, 4H), 2.71 (d, *J* = 7.7 Hz, 2H), 3.40 (d, *J* = 6.4 Hz, 4H), 3.63 (t, *J* = 6.3 Hz, 2H), 3.79 (s, 3H), 3.80 (s, 3H), 5.01 – 5.19 (m, 4H), 5.90 – 6.14 (m, 2H), 6.81 (s, 1H), 6.93 (d, *J* = 8.3 Hz, 1H), 7.07 (s, 1H), 7.10 (d, *J* = 2.3 Hz, 1H), 7.16 (dd, *J* = 8.3 Hz & 2.3 Hz, 1H) ppm.

**$^{13}$C-NMR (50 MHz, $CDCl_3$)** $\delta$ = 28.2, 32.4, 32.7, 36.5, 39.5, 45.0, 56.0, 56.0, 111.3, 112.2, 115.5, 115.5, 125.8, 127.8, 128.4, 129.3, 131.8 (2C), 132.8, 137.8, 137.9, 140.3, 155.6, 155.6 ppm.

**GC-MS** 386 (36, $M^+$), 385 (25, $M^+$), 384 (100, $M^+$), 312 (11), 307 (18), 293 (12), 266 (14), 265 (11), 252 (20), 251 (27), 235 (14), 221 (13), 219 (13), 207 (21), 165 (11), 153 (11), 132 (12), 131 (11), 115 (12)

**HRMS** $[M+H]^+$: calculated: 385.1929 Da, found: 385.1919 Da, difference: 1.0 mDa

(4-(5,5'-Diallyl-2,2'-dimethoxy-[1,1'-biphenyl]-4-yl)butyl)iodotriphenyl-λ$^5$-phosphane **15**

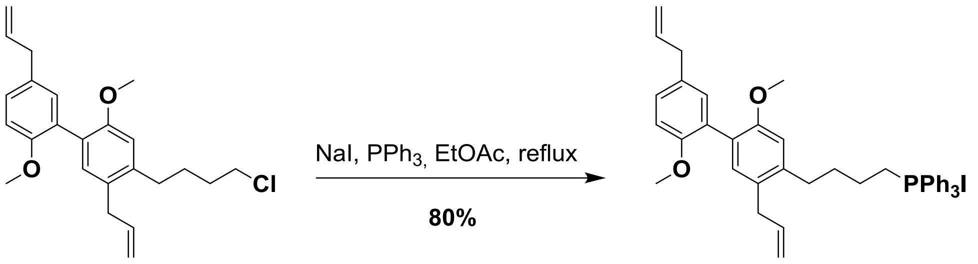


**14**
M = 384.94

**15**
M = 738.69

| | |
|---|---|
| **Procedure** | Chloride **14** (1050 mg, 2.73 mmol, 1 equiv.), NaI (409 mg, 2.73 mmol, 1 equiv.) and $PPh_3$ (715 mg, 2.73 mmol, 1 equiv.) were placed in a flask and EtOAc (13.6 mL) was added. The flask was flushed with argon and refluxed for 48 hours. Volatiles were removed *in vacuo*. To the residue $CHCl_3$ (12 mL) was added and the mixture was stirred at reflux temperature for 30 minutes. After filtration and evaporation of the solvent *in vacuo* the crude product was obtained as an oil. The oil was refluxed with ether (25 mL) for 1 hour. After cooling to room termperature the solution was poured off leaving a waxy material behind. This step was repeated with diethyl ether (25 mL) and heptane (10 mL). The waxy residue was dried under high vacuum which caused crystallization. The solid was crushed to obtain a colorless powder. |
| **Yield** | 80 % (1612 mg, 2.18 mmol) |
| **Appearance** | colorless powder |
| **Melting point** | 99.0 – 100.0 °C |
| **Sum formula** | $C_{42}H_{44}IO_2P$ |
| **$^1$H-NMR (400 MHz, $CDCl_3$)** | $\delta$ = 1.69 – 1.79 (m, 2H), 2.02 (p, *J* = 7.7 Hz, 2H), 2.64 (t, *J* = 7.6 Hz, 2H), 3.23 (d, *J* = 6.3 Hz, 2H), 3.34 (d, *J* = 6.8 Hz, 2H), 3.70 (s, 3H), 3.71 (s, 3H), 3.72 – 3.79 (m, 2H), 4.87 – 4.93 (m, 2H), 5.00 – 5.11 (m, 2H), 5.85 (ddt, *J* = 17.9 Hz & 9.3 Hz & 6.3 Hz, 1H), 5.96 (ddt, *J* = 16.8 Hz & 10.0 Hz & 6.8 Hz, 1H), 6.81 (s, 1H), 6.87 (d, *J* = 8.4 Hz, 1H), 6.94 (s, 1H), 7.00 (d, *J* = 2.3 Hz, 1H), 7.10 (dd, *J* = 8.3 Hz & 2.3 Hz, 1H), 7.65 – 7.71 (m, 6H), 7.75 – 7.83 (m, 9H) ppm. |
| **$^{13}$C-NMR (101 MHz, $CDCl_3$)** | $\delta$ = 22.5 (d, *J* = 4.3 Hz), 23.0, 23.5, 31.1 (d, *J* = 15.2 Hz), 32.1, 36.5, 39.5, 56.0, 56.5, 111.4, 112.5, 115.4, 115.5, 118.2 (d, *J* = 85.9 Hz, 3C), 125.7, 127.8, 128.4, 129.0, 130.6 (d, *J* = 12.5 Hz, 6C), 131.7, 131.8, 132.6, 133.8 (d, *J* = 10.0 Hz, 6C), 135.2 (d, *J* = 3.0 Hz, 3C), 137.9, 137.9, 139.5, 155.6 ppm. |
| **HRMS** | $[M-HI+H]^+$: calculated: 611.3073 Da, found: 611.3058 Da, difference: 1.5 mDa |

(*Z*)-5,5'-Diallyl-4-(6-(5'-allyl-2',6-dimethoxy-[1,1'-biphenyl]-3-yl)hex-4-en-1-yl)-2,2'-dimethoxy-1,1'-biphenyl **16**

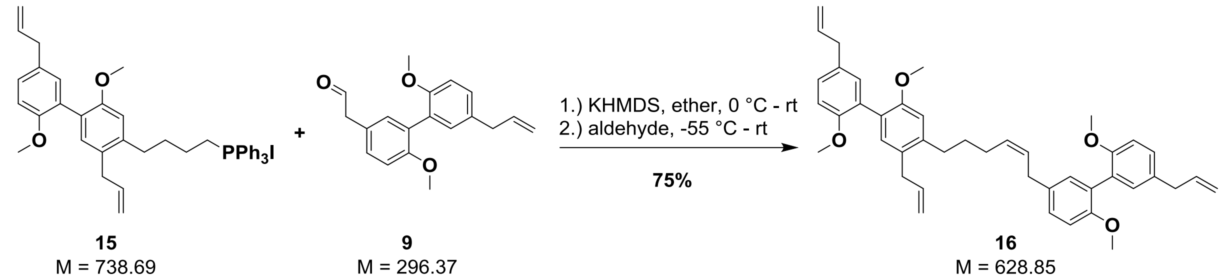

| | |
|---|---|
| **Procedure** | Phosphonium salt **15** (30 mg, 0.041 mmol, 1 equiv.) was placed in an oven dried flask. The flask was closed with a septum and was evacuated and flushed with argon on the schlenck line three times. Dry diethyl ether (1 mL) was added. The mixture was cooled to 0 °C. KHMDS (81 µL, 0.041 mmol, 1 equiv., 0.5M in toluene) was added by syringe. Then the reaction was stirred for 5 minutes at 0 °C and 20 minutes at room temperature. After cooling the solution to -55 °C aldehyde **9** (12 mg, 0.041 mmol, 1 equiv.) was added as a solution in dry diethyl ether and the reaction was allowed to reach room temperature and was stirred at room temperature for 1 hour. $H_2O$ (1 mL) was added and the mixture was extracted with EtOAc (3 x 1 mL). The organic phase was dried over $MgSO_4$. After evaporation of the solvent *in vacuo* purification by preparative TLC (PE/EtOAc = 5/1) the product was obtained as an insaparable isomeric mixture. |
| **Yield** | 75 % (19 mg, 0.03 mmol, purity > 87.5%) |
| **Appearance** | colorless oil |
| **Sum formula** | $C_{43}H_{48}O_4$ |
| **TLC** | $R_f$ (LP/EtOAc = 5/1) = 0.45 |
| **$^1$H-NMR (600 MHz, CDCl$_3$)** | $\delta$ = 1.74 (p, *J* = 7.9 Hz, 2H), 2.27 (q, *J* = 7.3 Hz, 2H), 2.66 (d, *J* = 7.8 Hz, 2H), 3.33 – 3.37 (m, 6H), 3.41 (d, *J* = 7.4 Hz, 2H), 3.74 (s, 3H), 3.75 (s, 3H), 3.75 (s, 3H), 3.75 (s, 3H), 4.99 – 5.12 (m, 6H), 5.53 – 5.69 (m, 2H), 5.90 – 6.04 (m, 3H), 6.76 (s, 1H), 6.88 – 6.92 (m, 3H), 7.01 (s, 1H), 7.07 (m, 3H), 7.10 – 7.16 (m, 3H) ppm. |
| **$^{13}$C-NMR (151 MHz, CDCl$_3$)** | $\delta$ = 27.6, 31.1, 32.8, 33.0, 36.6, 39.5, 39.6, 56.0, 56.0, 56.0, 56.1, 111.3, 111.3, 111.4, 112.3, 115.5, 115.6, 115.6, 125.6, 127.9, 128.0, 128.0, 128.3, 128.4, 128.5, 129.2, 129.4, 130.2, 131.5, 131.8, 131.9, 131.9 (2C), 132.7, 132.9, 138.0, 138.0, 138.0, 141.0, 155.5, 155.6, 155.6, 155.7 ppm. |
| **HRMS** | $[M+Na]^+$: calculated: 651.3445 Da, found: 651.3416 Da, difference: 2.9 mDa<br>$[M+K]^+$: calculated: 667.3184 Da, found: 667.3160 Da, difference: 2.4 mDa |

Magnolol dimer (**1**)

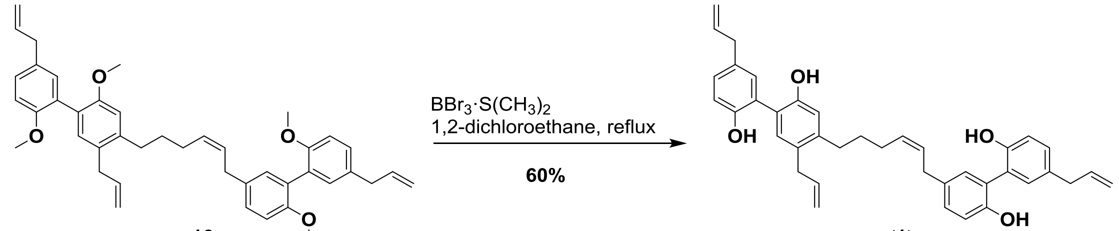


**16**
M = 628.85

(**1**)
M = 572.75

| | |
|---|---|
| **Procedure** | Tetramethylated magnolol dimer **16** (19 mg, 0.03 mmol, 1 equiv.), 1,2-dichloroethane (1 mL) and $BBr_3 \cdot S(CH_3)_2$ complex (48 mg, 0.15 mmol, 5 equiv.) were placed in an oven dried vial. The vial was flushed with argon and closed tighlty with a screw cap. The mixture was refluxed (thermoblock was set to 91 °C) for 4.5 hours. $H_2O$ (2 mL) and 1,2-dichloroethane (1 mL) was added and phases were separated. Organic phase was extracted with 1,2-dichloroethane (2 x 1 mL) and the combined organic phase was washed with brine and dried over $MgSO_4$. After evaporation of the solvent and purification by preparative HPLC the product was obtained. |
| **Yield** | 60 % (10.5 mg, 0.018 mmol) |
| **Appearance** | colorless oil |
| **Sum formula** | $C_{39}H_{40}O_4$ |
| **TLC** | $R_f$ (DCM/MeOH = 10/1) = 0.8 |
| **$^1$H-NMR (400 MHz, $CDCl_3$)** | $\delta$ = 1.61 – 1.73 (m, 4H), 2.22 (q, $J$ = 7.3 Hz, 2H), 2.59 (d, $J$ = 7.9 Hz, 2H), 3.29 – 3.42 (m, 8H), 4.95 – 5.12 (m, 6H), 5.53 – 5.73 (m, 3H), 5.86 – 6.03 (m, 4H), 6.75 (s, 1H), 6.95 (dd, $J$ = 8.2 Hz & 1.6 Hz, 3H), 7.01 (s, 1H), 7.07 (dd, $J$ = 6.3 Hz & 2.2 Hz, 2H), 7.09 – 7.14 (m, 4H) ppm. |
| **$^{13}$C-NMR (101 MHz, $CDCl_3$)** | $\delta$ = 27.3, 30.7, 32.2, 32.7, 36.5, 39.5, 39.5, 115.8, 115.9, 116.0, 116.8, 116.9, 116.9, 117.1, 121.5, 123.9, 124.0 (2C), 128.8, 129.9, 129.9, 130.1, 130.7, 131.0, 131.0 (2C), 131.3, 132.4, 133.2, 133.4, 134.4, 137.5, 137.6, 137.7, 142.6, 151.1, 151.1, 151.3, 151.4 ppm. |
| **HRMS** | $[M-H]^-$: calculated: 571.2854 Da, found: 571.2862 Da, difference: 0.8 mDa |

# NMR spectra

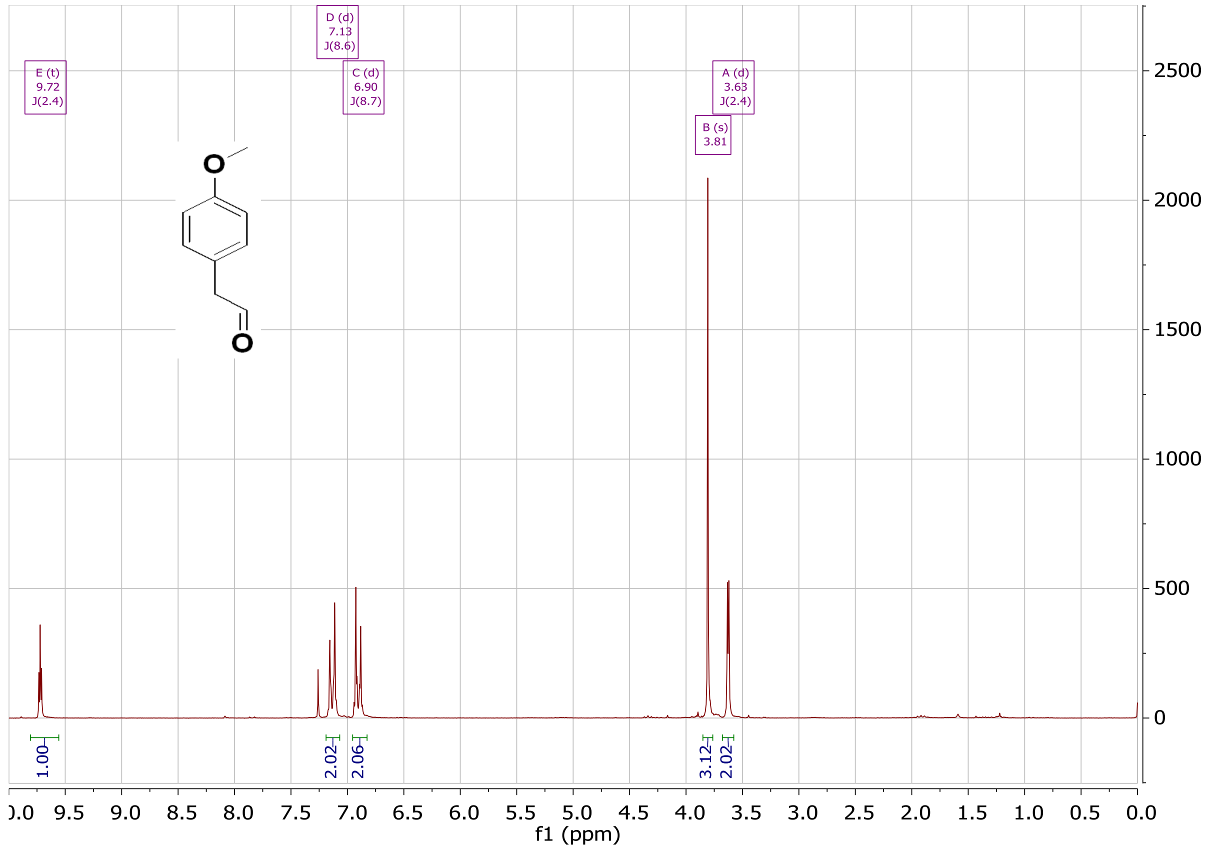
E (t)
9.72
J(2.4)
D (d)
7.13
J(8.6)
C (d)
6.90
J(8.7)
A (d)
3.63
J(2.4)
B (s)
3.81
1.00
2.02
2.06
3.12
2.02
f1 (ppm)

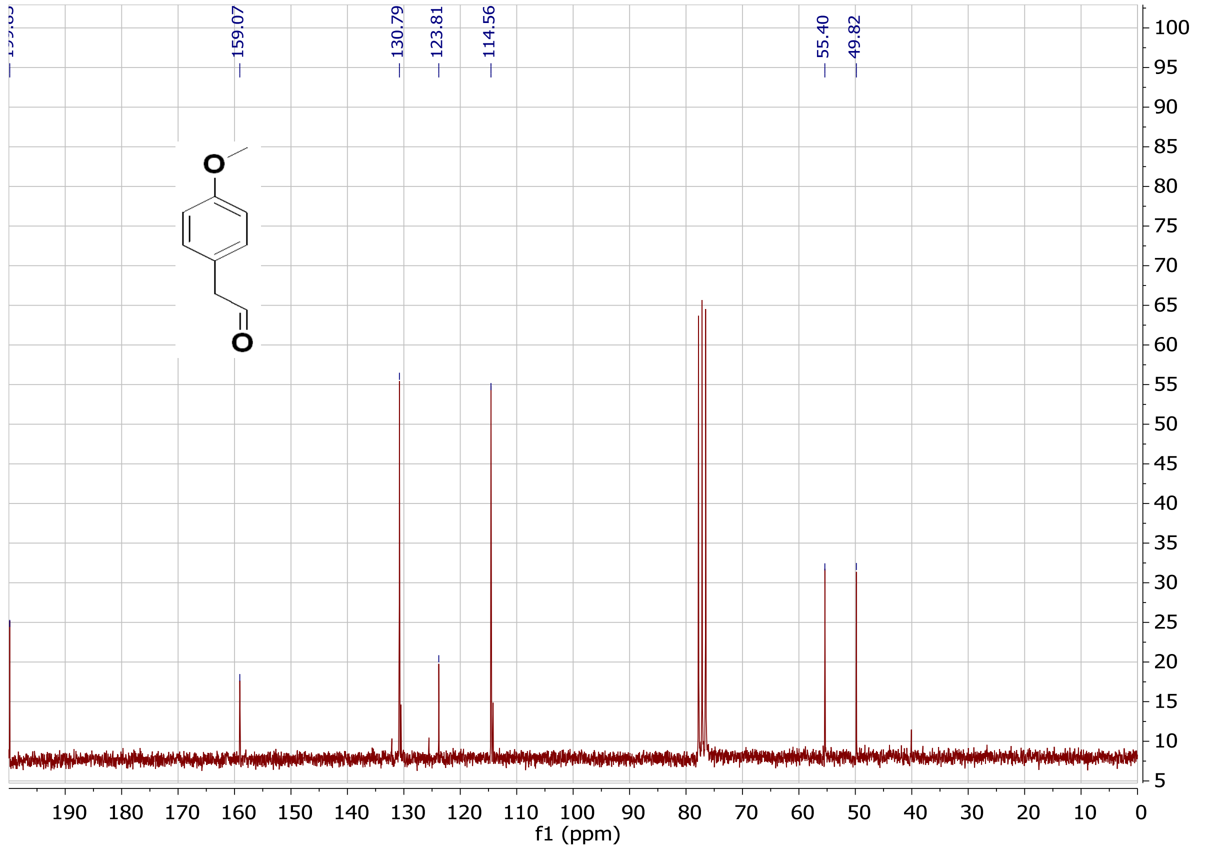
159.07
130.79
123.81
114.56
55.40
49.82
f1 (ppm)

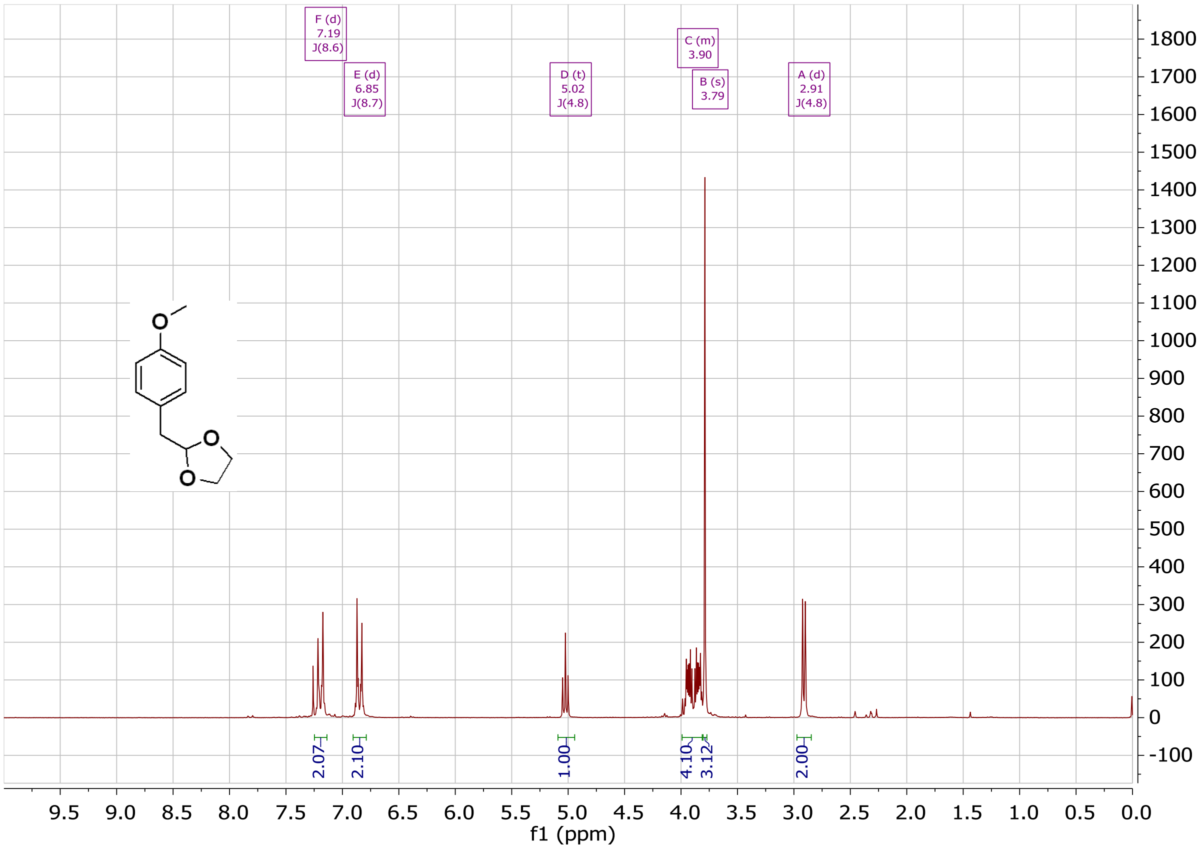

F (d)
7.19
J(8.6)
E (d)
6.85
J(8.7)
D (t)
5.02
J(4.8)
C (m)
3.90
B (s)
3.79
A (d)
2.91
J(4.8)
2.07
2.10
1.00
4.10
3.12
2.00
f1 (ppm)


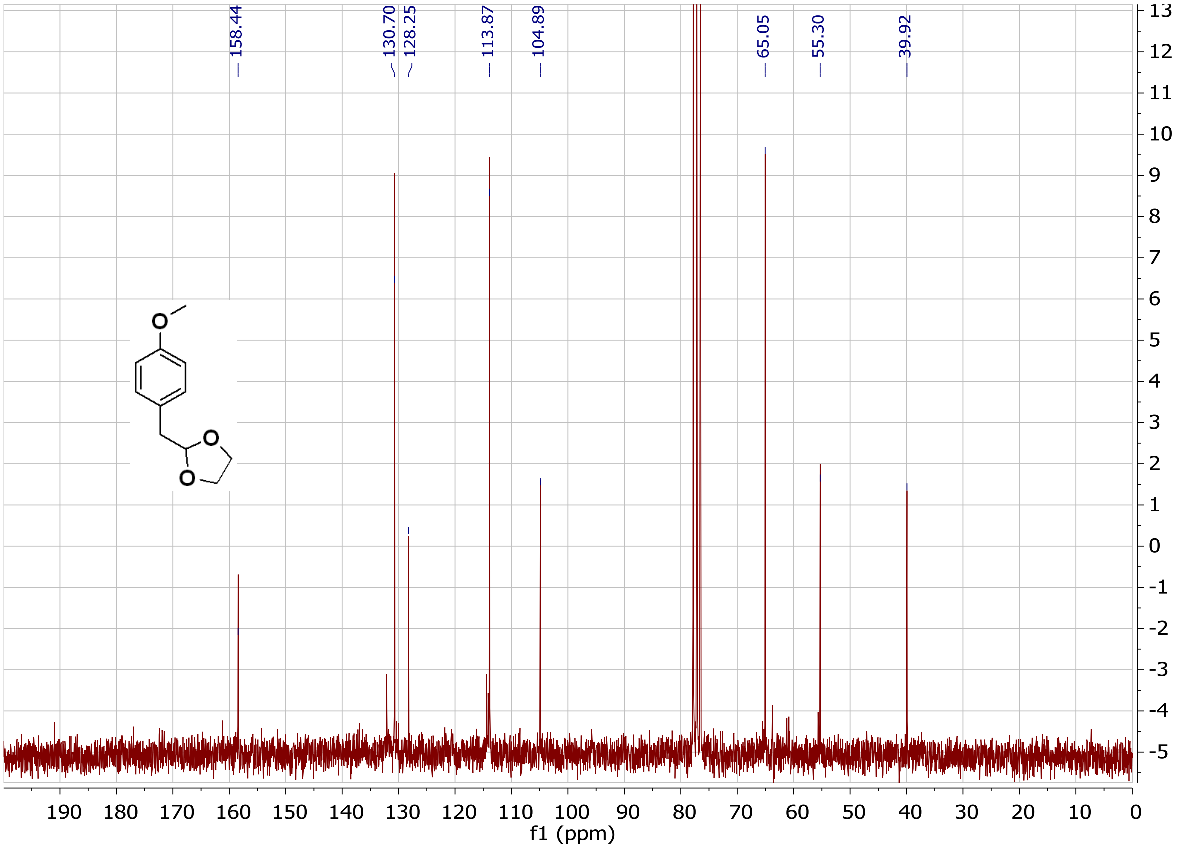

158.44
130.70
128.25
113.87
104.89
65.05
55.30
39.92
f1 (ppm)

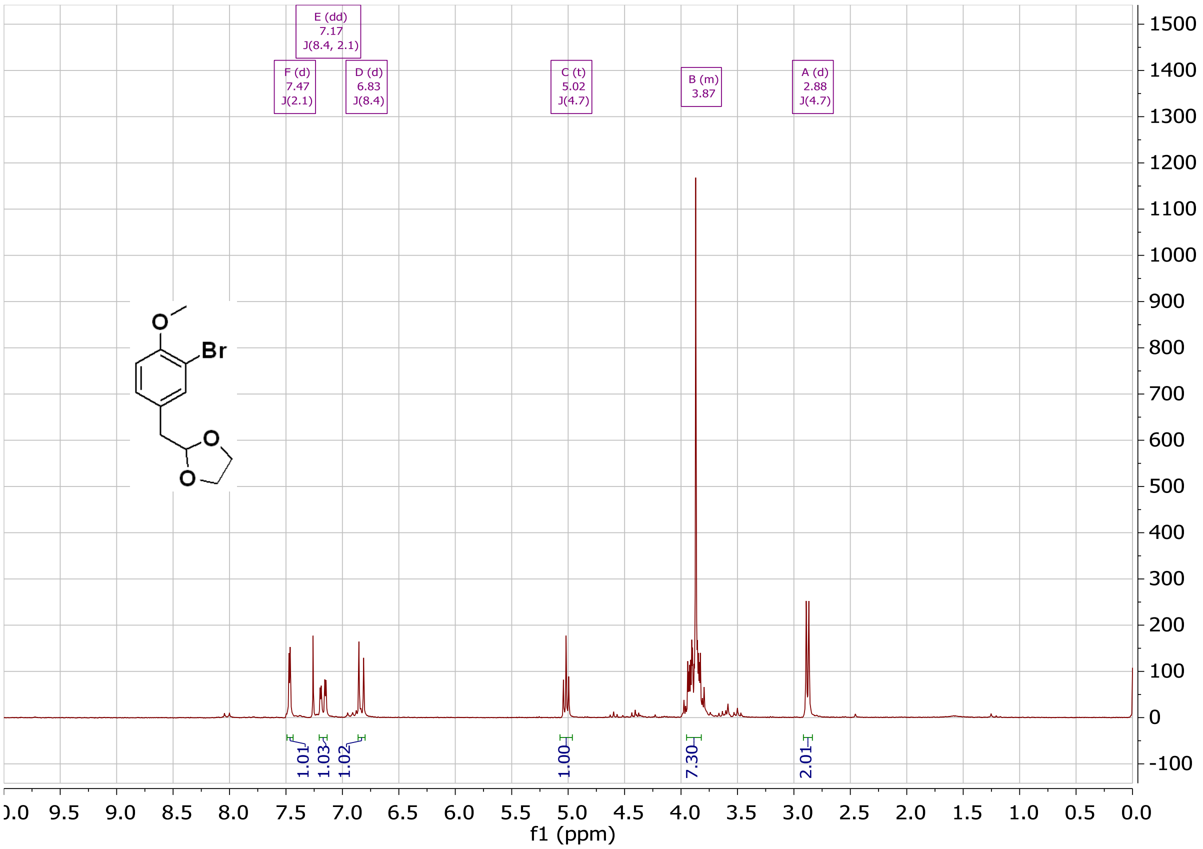
E (dd)
7.17
J(8.4, 2.1)
F (d)
7.47
J(2.1)
D (d)
6.83
J(8.4)
C (t)
5.02
J(4.7)
B (m)
3.87
A (d)
2.88
J(4.7)
1.01
1.03
1.02
1.00
7.30
2.01
Br
O
f1 (ppm)

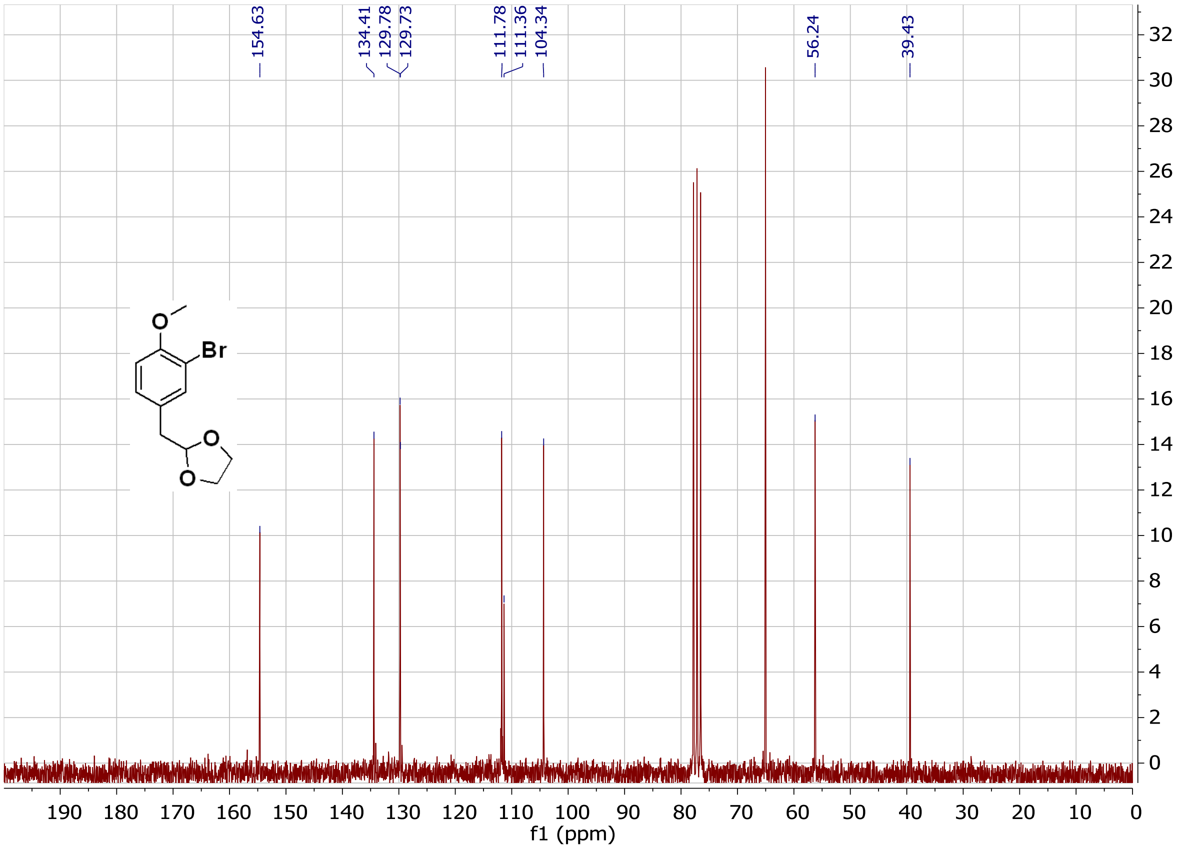
154.63
134.41
129.78
129.73
111.78
111.36
104.34
56.24
39.43
Br
O
f1 (ppm)

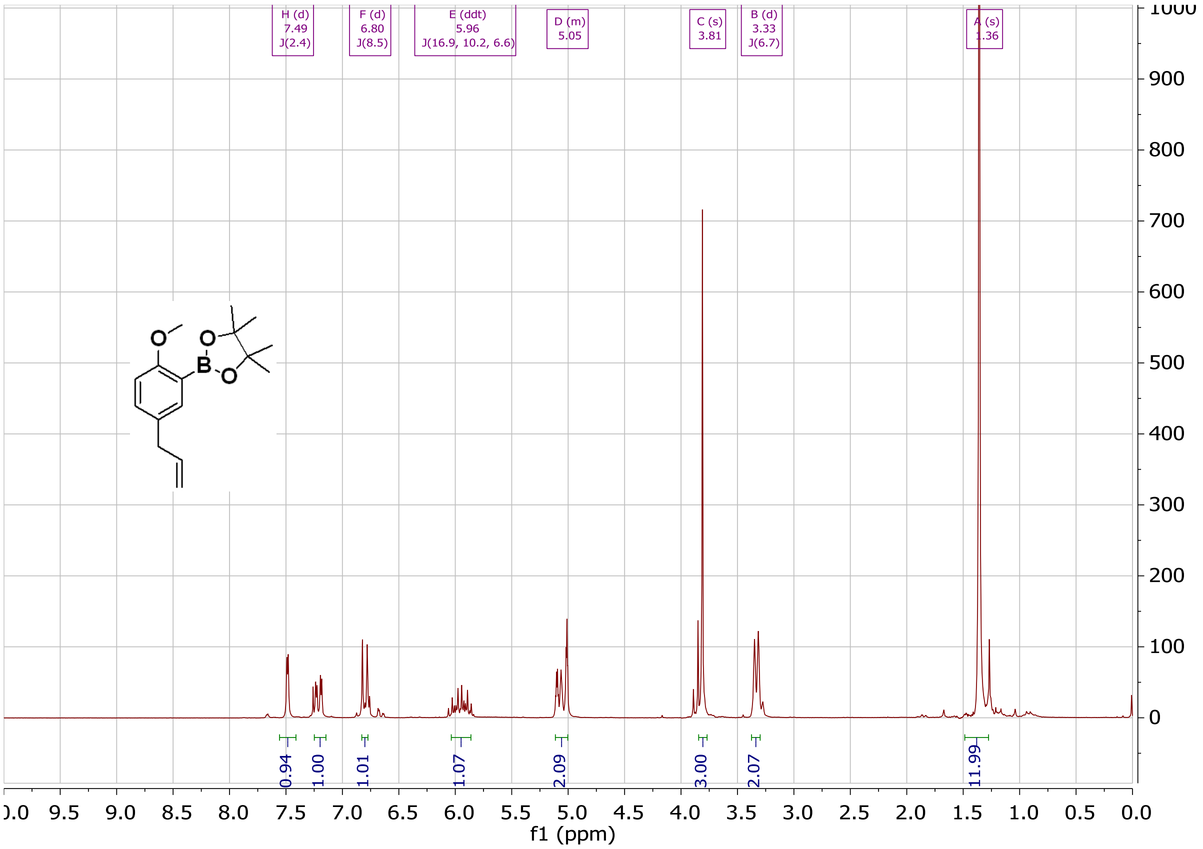
H (d)
7.49
J(2.4)
F (d)
6.80
J(8.5)
E (ddt)
5.96
J(16.9, 10.2, 6.6)
D (m)
5.05
C (s)
3.81
B (d)
3.33
J(6.7)
A (s)
1.36
0.94
1.00
1.01
1.07
2.09
3.00
2.07
11.99
f1 (ppm)

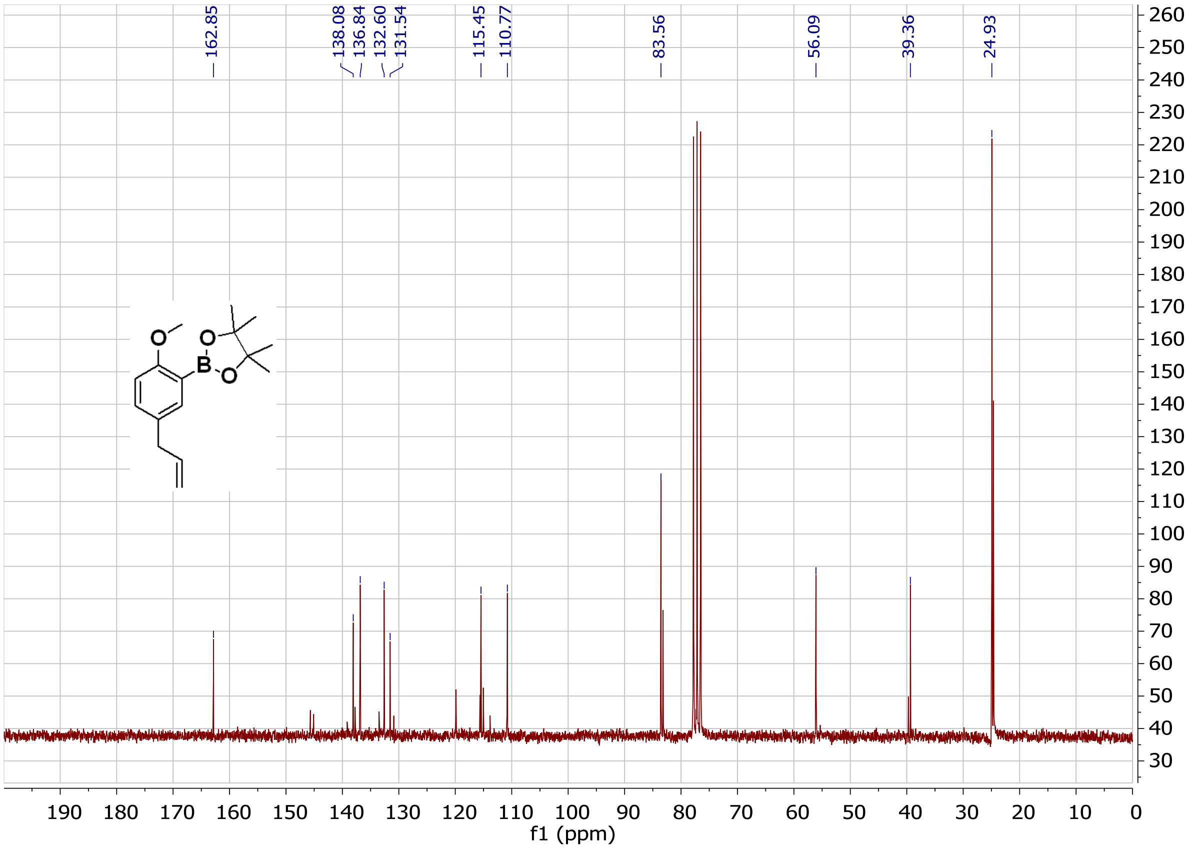
162.85
138.08
136.84
132.60
131.54
115.45
110.77
83.56
56.09
39.36
24.93
f1 (ppm)

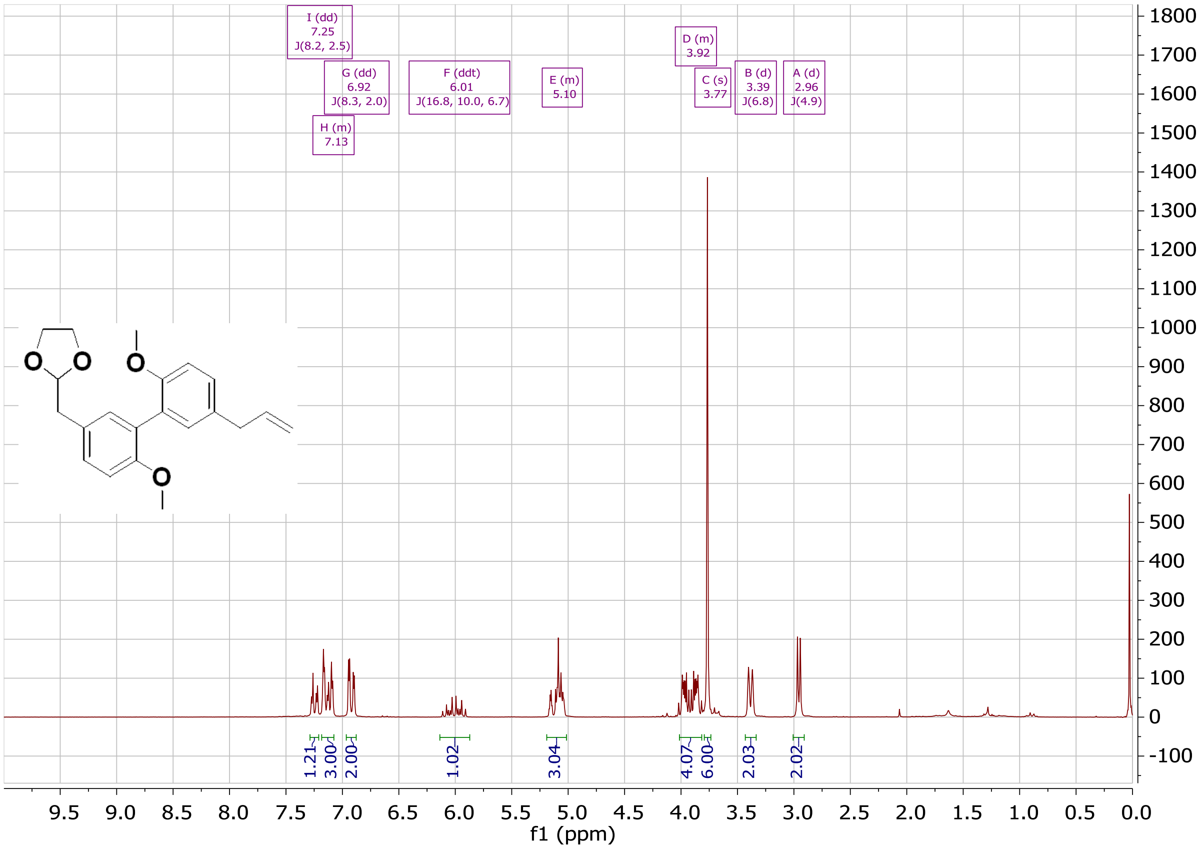
I (dd)
7.25
J(8.2, 2.5)
G (dd)
6.92
J(8.3, 2.0)
H (m)
7.13
F (ddt)
6.01
J(16.8, 10.0, 6.7)
E (m)
5.10
D (m)
3.92
C (s)
3.77
B (d)
3.39
J(6.8)
A (d)
2.96
J(4.9)
1.21
3.00
2.00
1.02
3.04
4.07
6.00
2.03
2.02
f1 (ppm)

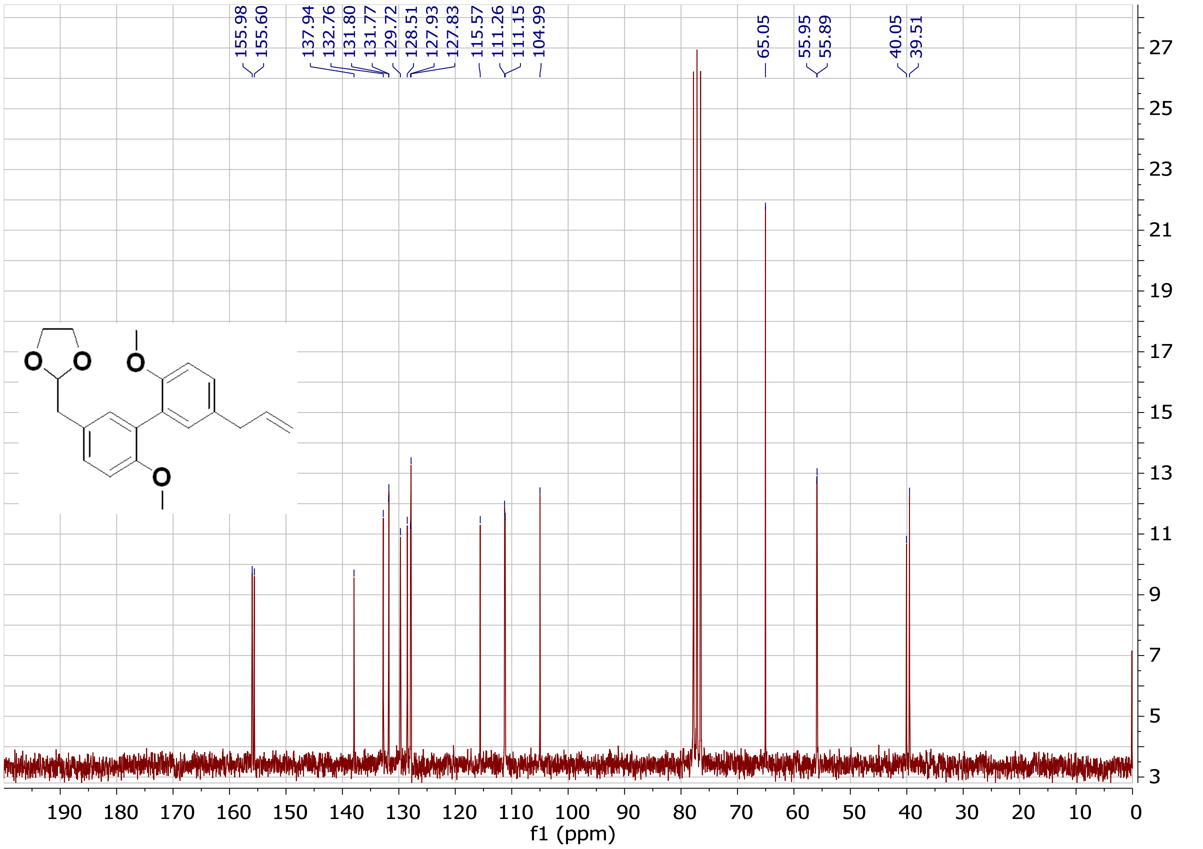
155.98
155.60
137.94
132.76
131.80
131.77
129.72
128.51
127.93
127.83
115.57
111.26
111.15
104.99
65.05
55.95
55.89
40.05
39.51
f1 (ppm)

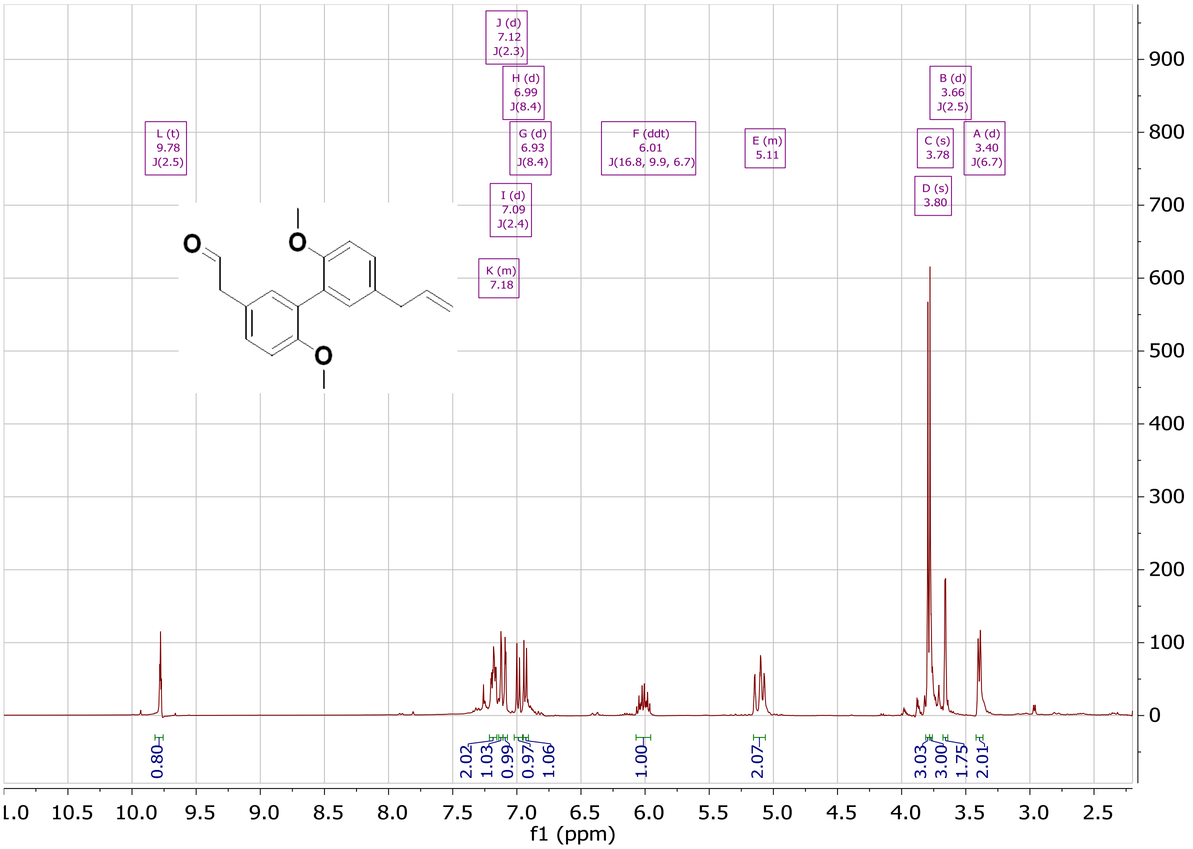
J (d) 7.12 J(2.3)
H (d) 6.99 J(8.4)
L (t) 9.78 J(2.5)
G (d) 6.93 J(8.4)
F (ddt) 6.01 J(16.8, 9.9, 6.7)
E (m) 5.11
B (d) 3.66 J(2.5)
C (s) 3.78
A (d) 3.40 J(6.7)
D (s) 3.80
I (d) 7.09 J(2.4)
K (m) 7.18
0.80
2.02
1.03
0.99
0.97
1.06
1.00
2.07
3.03
3.00
1.75
2.01
f1 (ppm)

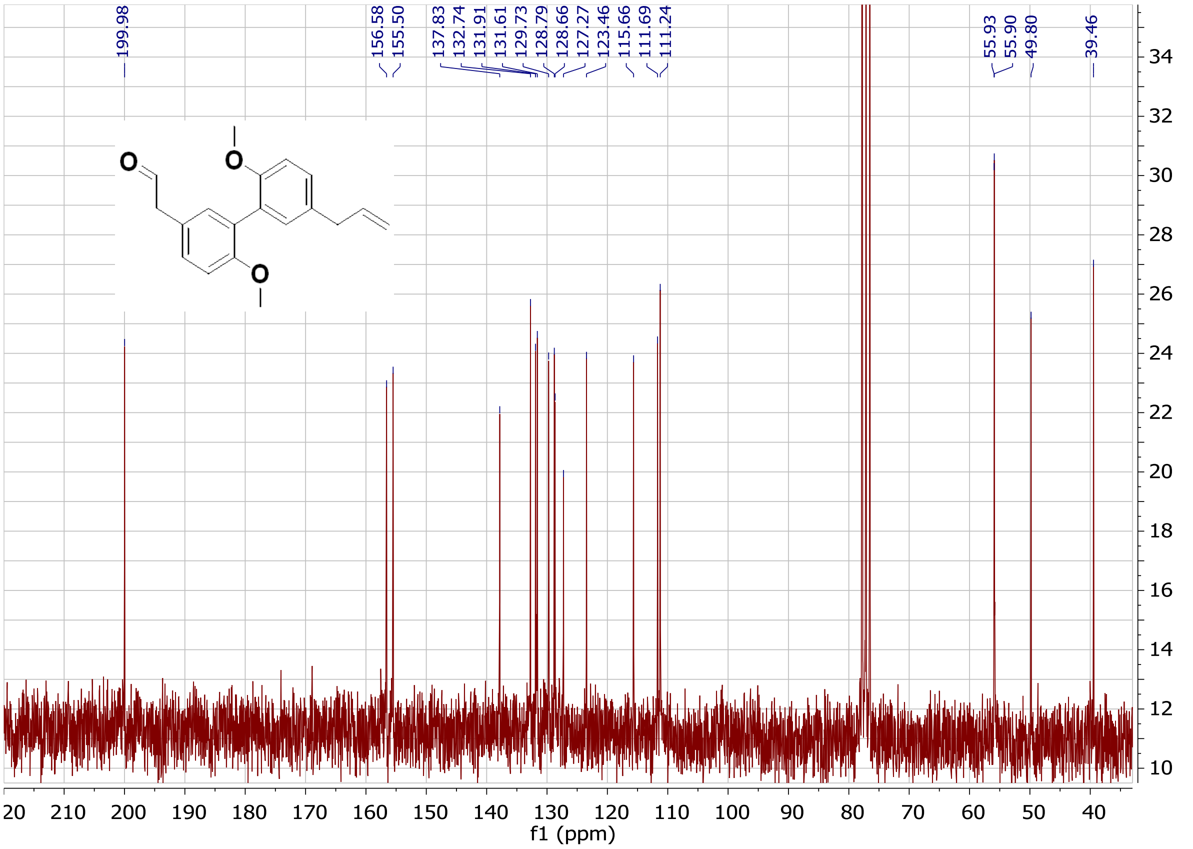
199.98
156.58
155.50
137.83
132.74
131.91
131.61
129.73
128.79
128.66
127.27
123.46
115.66
111.69
111.24
55.93
55.90
49.80
39.46
f1 (ppm)

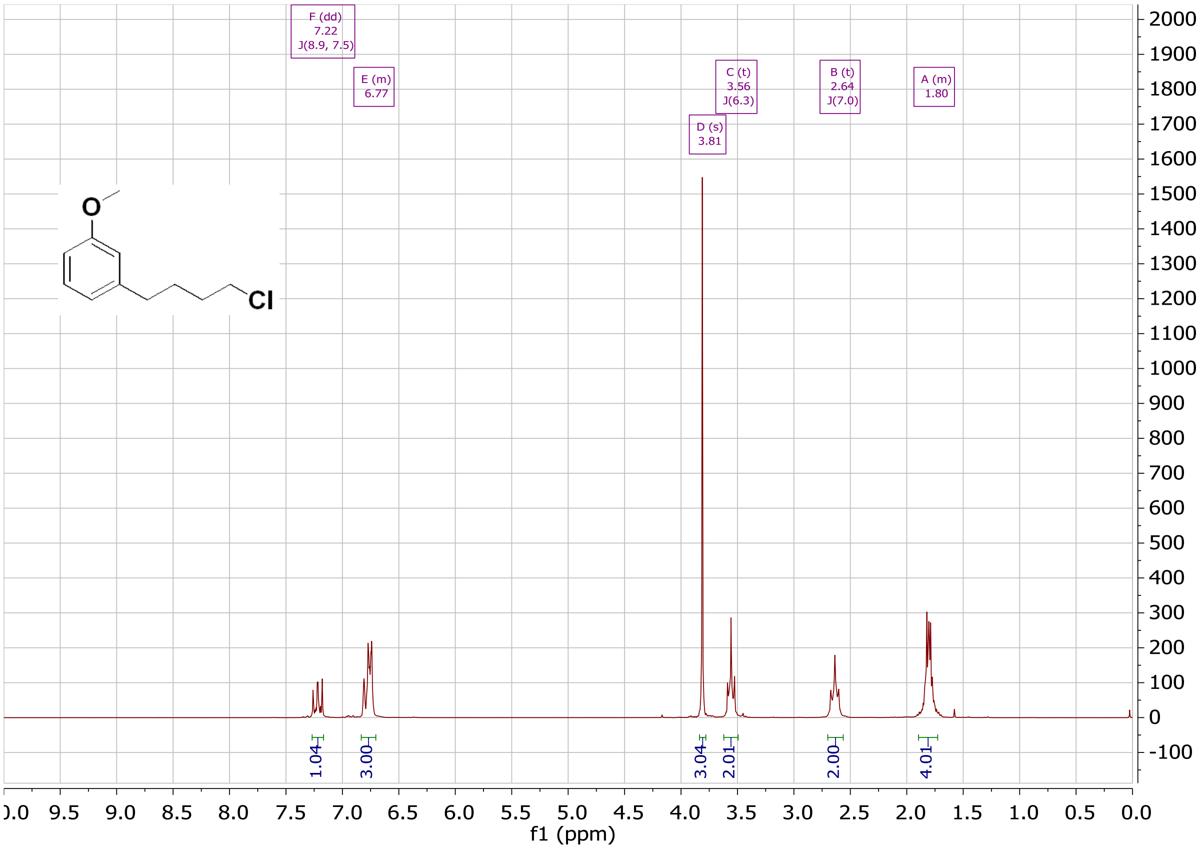
F (dd)
7.22
J(8.9, 7.5)
E (m)
6.77
C (t)
3.56
J(6.3)
B (t)
2.64
J(7.0)
A (m)
1.80
D (s)
3.81
O
Cl
1.04
3.00
3.04
2.01
2.00
4.01
f1 (ppm)

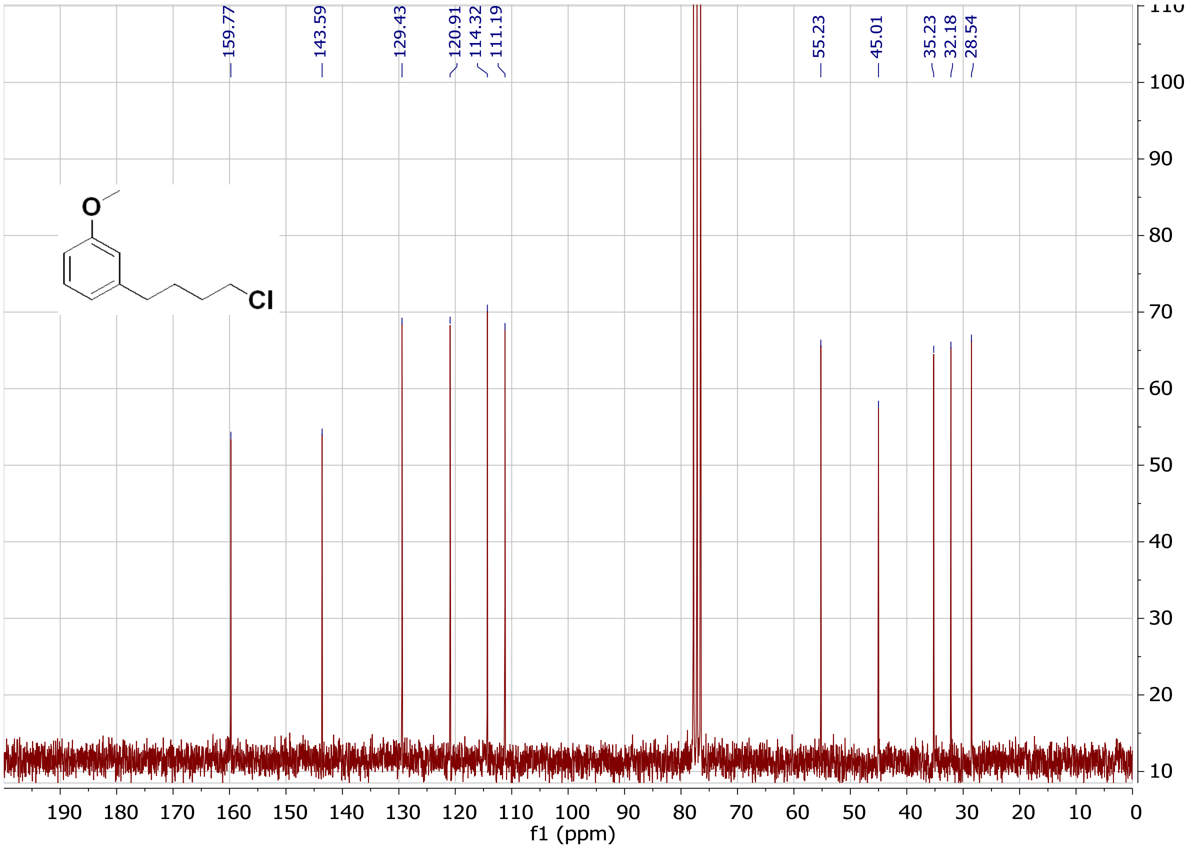
159.77
143.59
129.43
120.91
114.32
111.19
55.23
45.01
35.23
32.18
28.54
O
Cl
f1 (ppm)

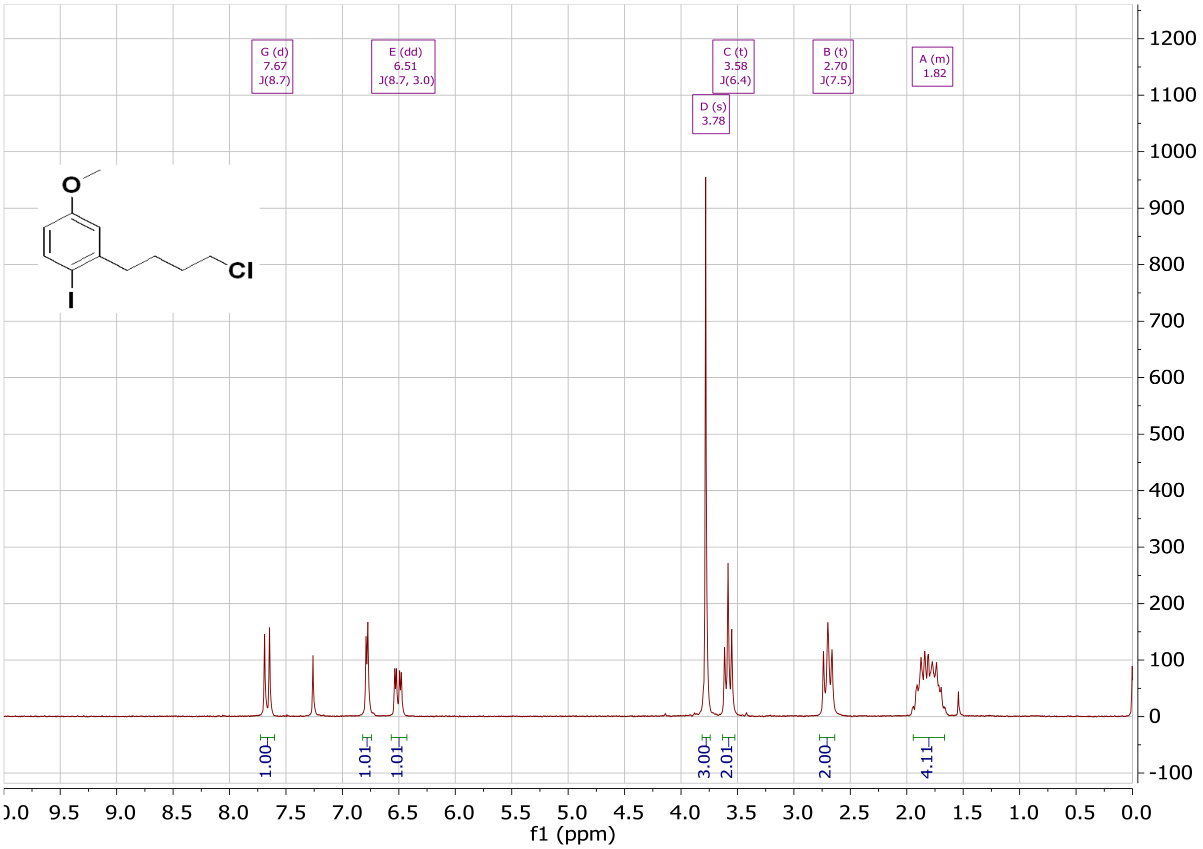
G (d)
7.67
J(8.7)
E (dd)
6.51
J(8.7, 3.0)
D (s)
3.78
C (t)
3.58
J(6.4)
B (t)
2.70
J(7.5)
A (m)
1.82
O
Cl
I
1.00
1.01
1.01
3.00
2.01
2.00
4.11
f1 (ppm)

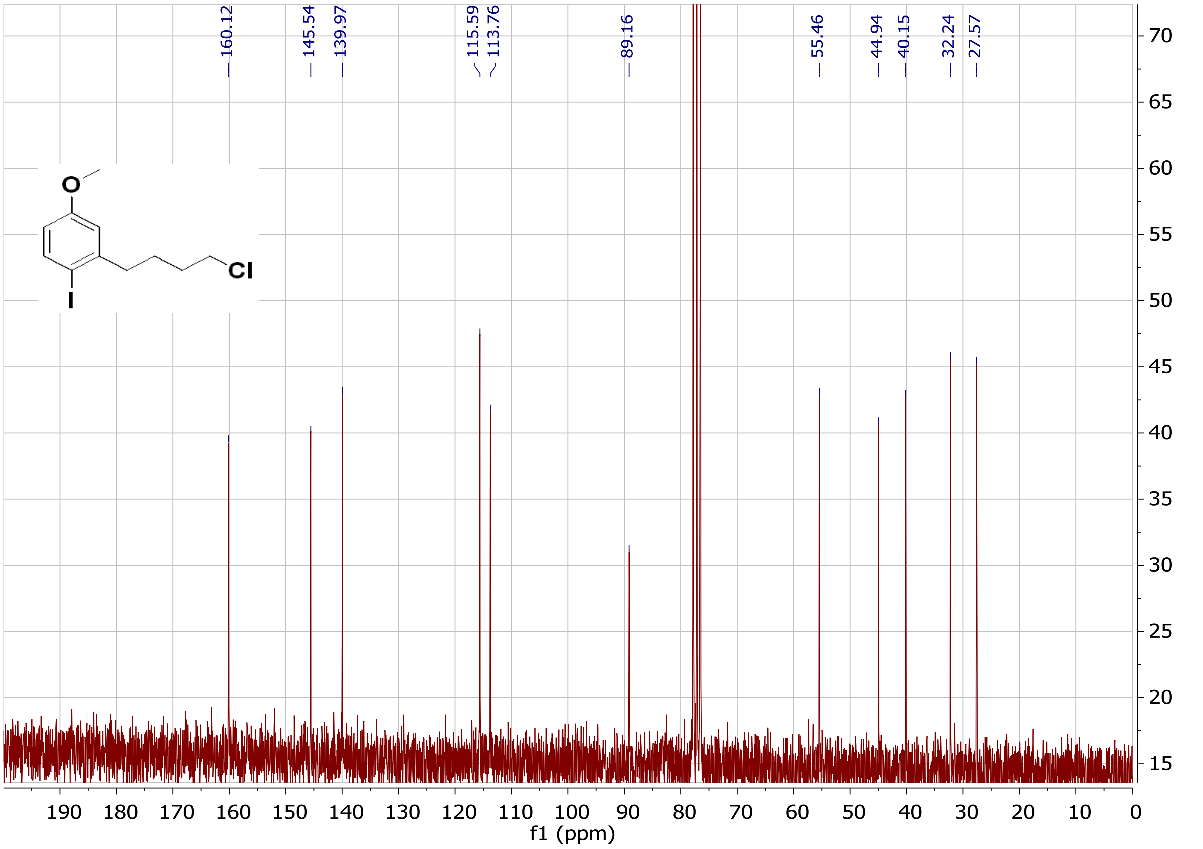
160.12
145.54
139.97
115.59
113.76
89.16
55.46
44.94
40.15
32.24
27.57
O
Cl
I
f1 (ppm)

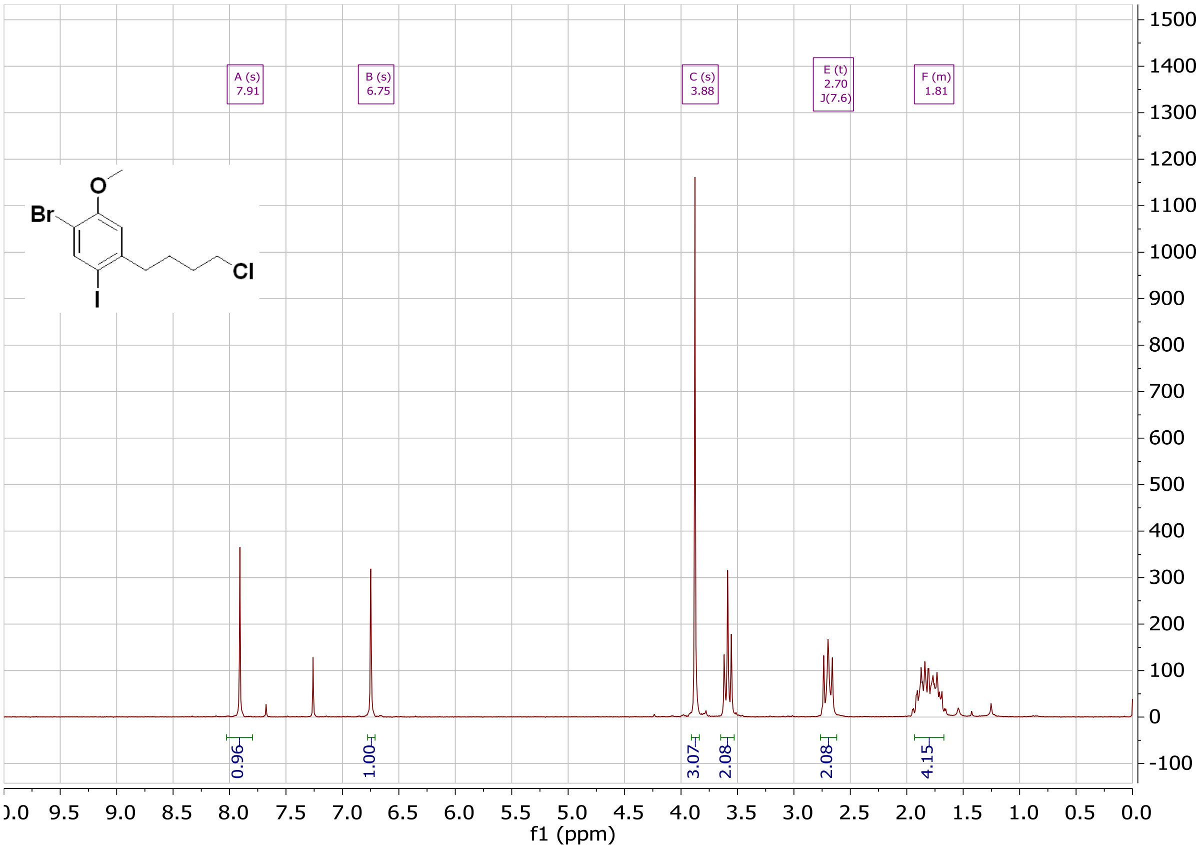

A (s) 7.91
B (s) 6.75
C (s) 3.88
E (t) 2.70 J(7.6)
F (m) 1.81
0.96
1.00
3.07
2.08
2.08
4.15
Br
O
I
Cl
f1 (ppm)


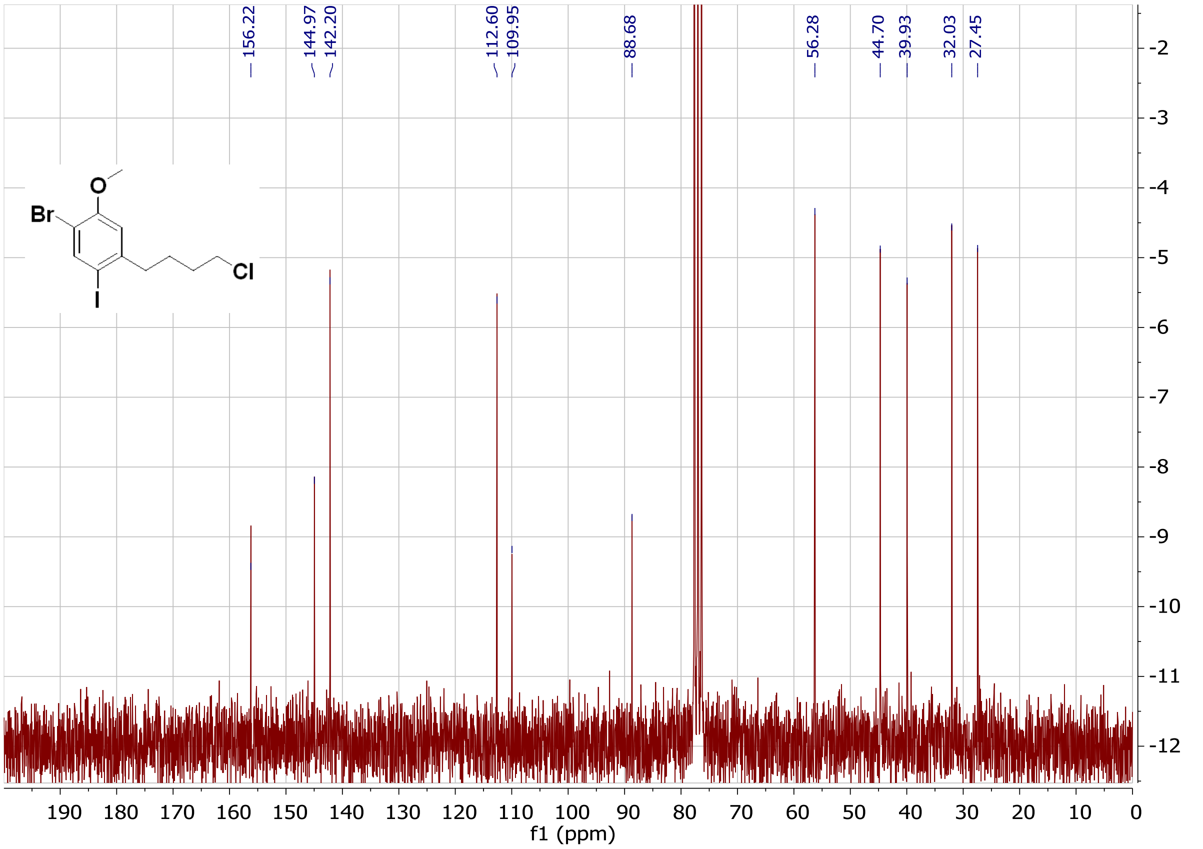

156.22
144.97
142.20
112.60
109.95
88.68
56.28
44.70
39.93
32.03
27.45
Br
O
I
Cl
f1 (ppm)

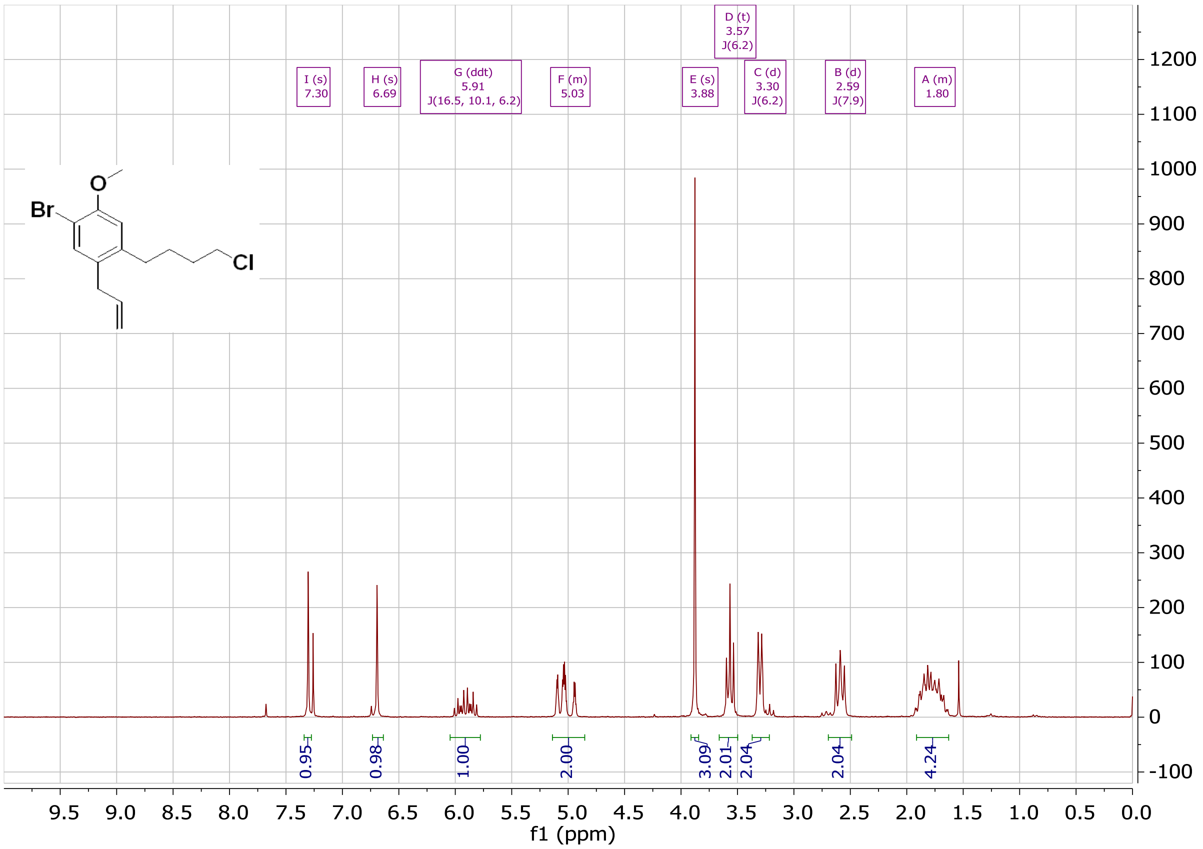

I (s)
7.30
H (s)
6.69
G (ddt)
5.91
J(16.5, 10.1, 6.2)
F (m)
5.03
E (s)
3.88
D (t)
3.57
J(6.2)
C (d)
3.30
J(6.2)
B (d)
2.59
J(7.9)
A (m)
1.80
Br
O
Cl
0.95
0.98
1.00
2.00
3.09
2.01
2.04
2.04
4.24
f1 (ppm)


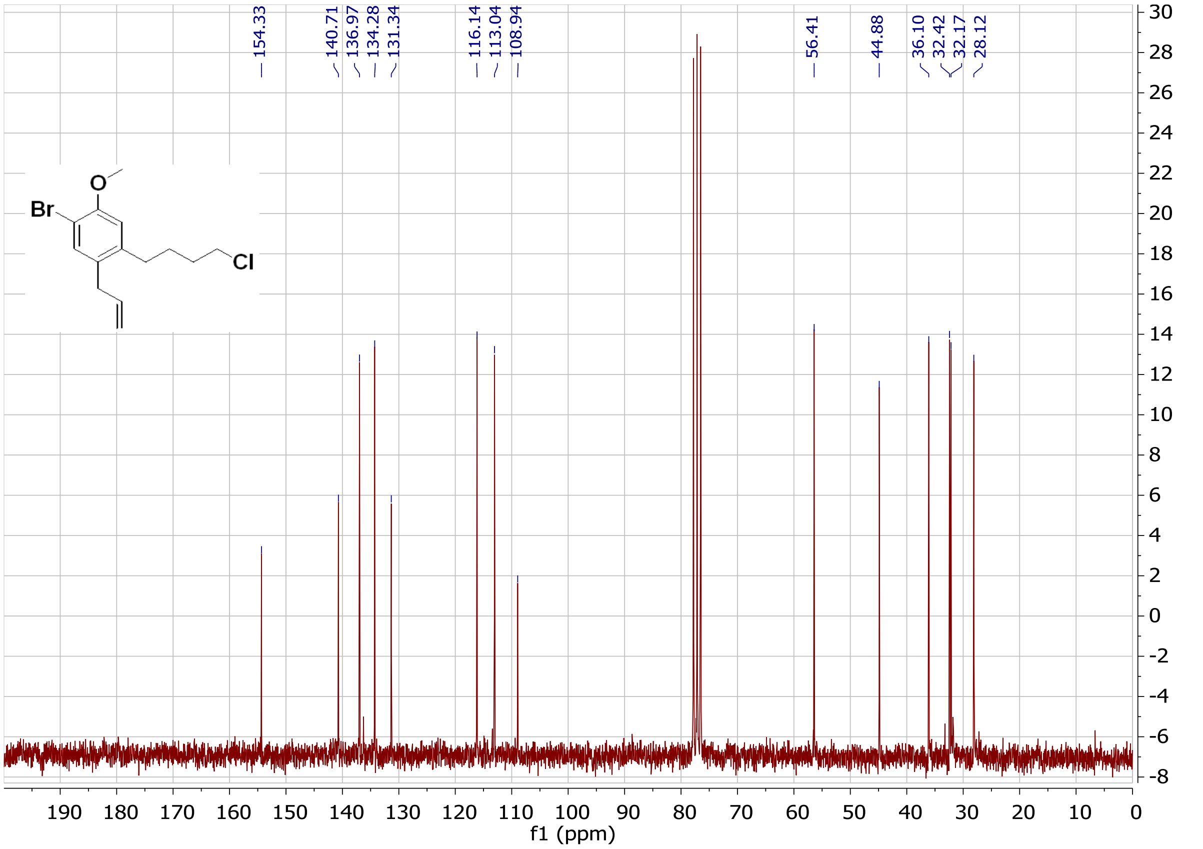

154.33
140.71
136.97
134.28
131.34
116.14
113.04
108.94
56.41
44.88
36.10
32.42
32.17
28.12
Br
O
Cl
f1 (ppm)

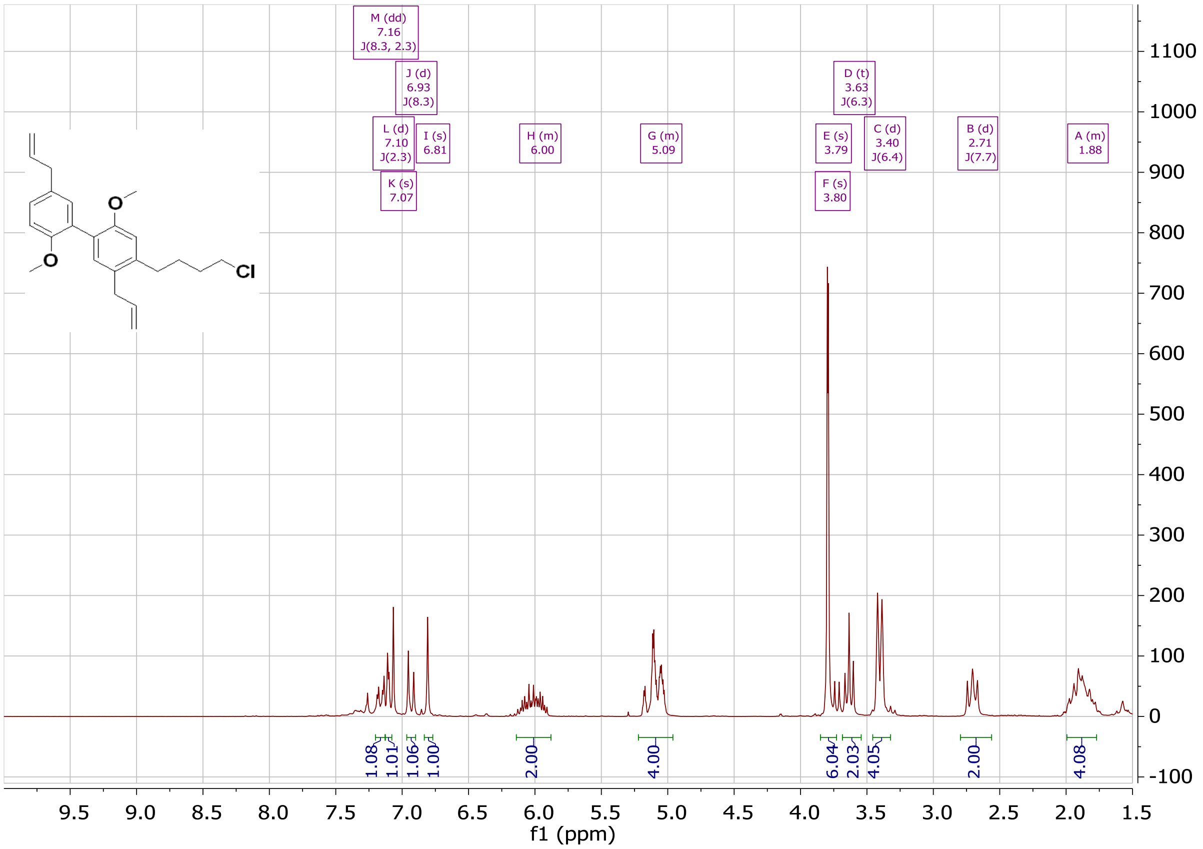

M (dd) 7.16 J(8.3, 2.3)
J (d) 6.93 J(8.3)
L (d) 7.10 J(2.3)
I (s) 6.81
K (s) 7.07
H (m) 6.00
G (m) 5.09
D (t) 3.63 J(6.3)
E (s) 3.79
F (s) 3.80
C (d) 3.40 J(6.4)
B (d) 2.71 J(7.7)
A (m) 1.88
O
O
Cl
1.08
1.01
1.06
1.00
2.00
4.00
6.04
2.03
4.05
2.00
4.08
f1 (ppm)


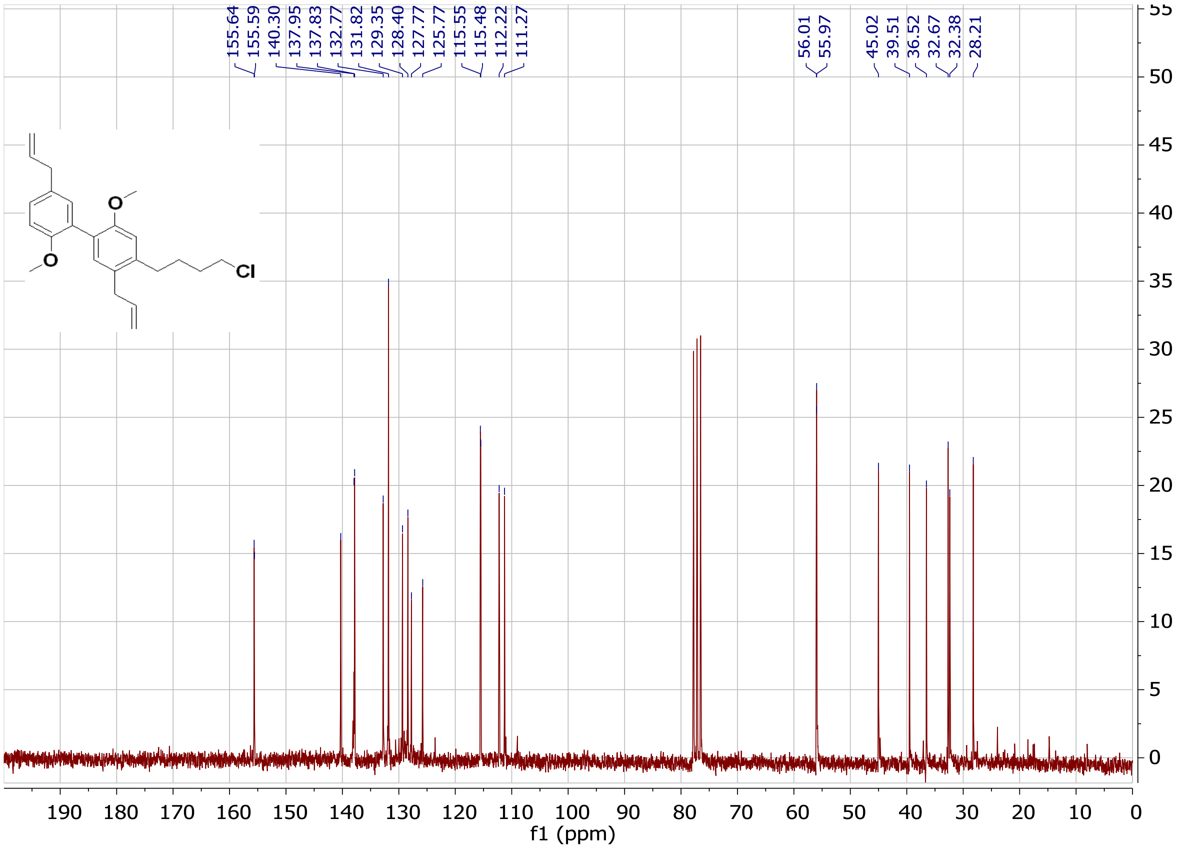

155.64
155.59
140.30
137.95
137.83
132.77
131.82
129.35
128.40
127.77
125.77
115.55
115.48
112.22
111.27
56.01
55.97
45.02
39.51
36.52
32.67
32.38
28.21
O
O
Cl
f1 (ppm)

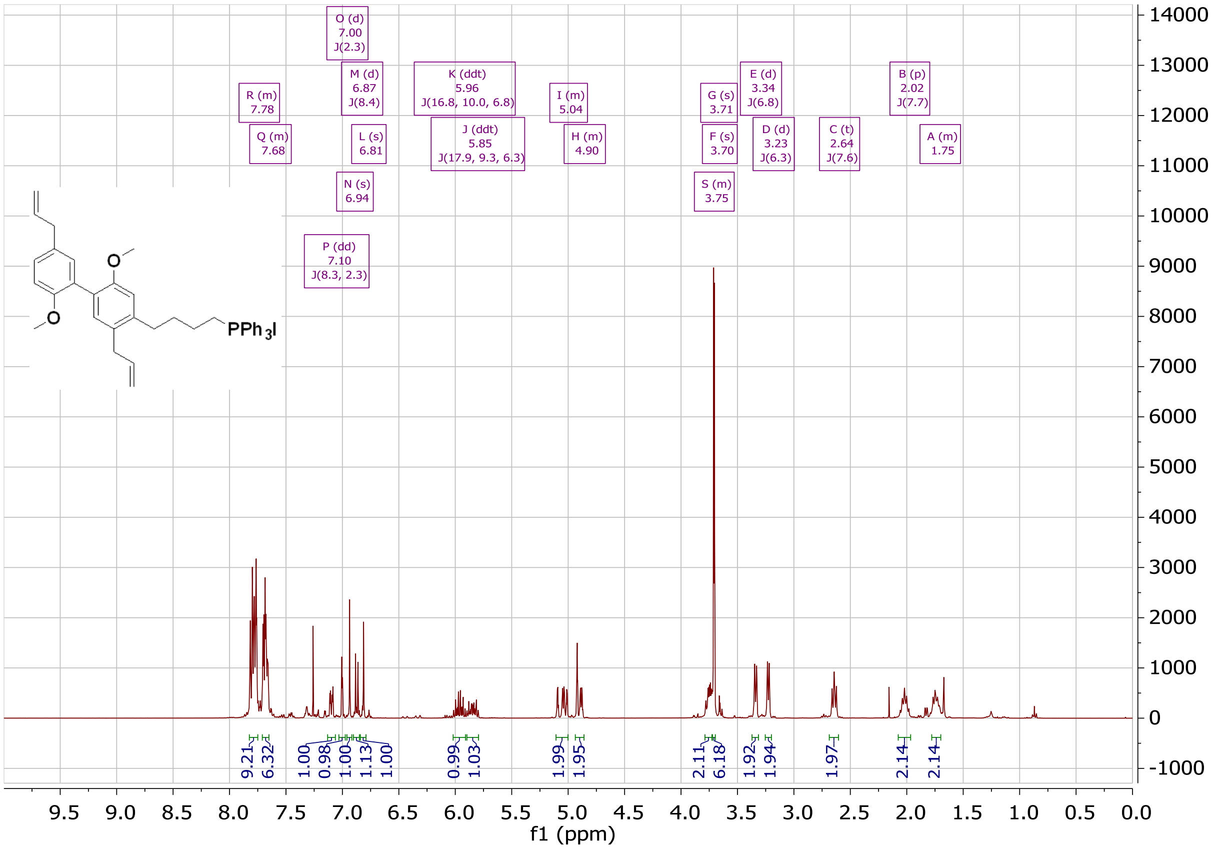

R (m) 7.78
Q (m) 7.68
O (d) 7.00 J(2.3)
M (d) 6.87 J(8.4)
L (s) 6.81
N (s) 6.94
P (dd) 7.10 J(8.3, 2.3)
K (ddt) 5.96 J(16.8, 10.0, 6.8)
J (ddt) 5.85 J(17.9, 9.3, 6.3)
I (m) 5.04
H (m) 4.90
G (s) 3.71
F (s) 3.70
S (m) 3.75
E (d) 3.34 J(6.8)
D (d) 3.23 J(6.3)
C (t) 2.64 J(7.6)
B (p) 2.02 J(7.7)
A (m) 1.75
PPh3I
f1 (ppm)


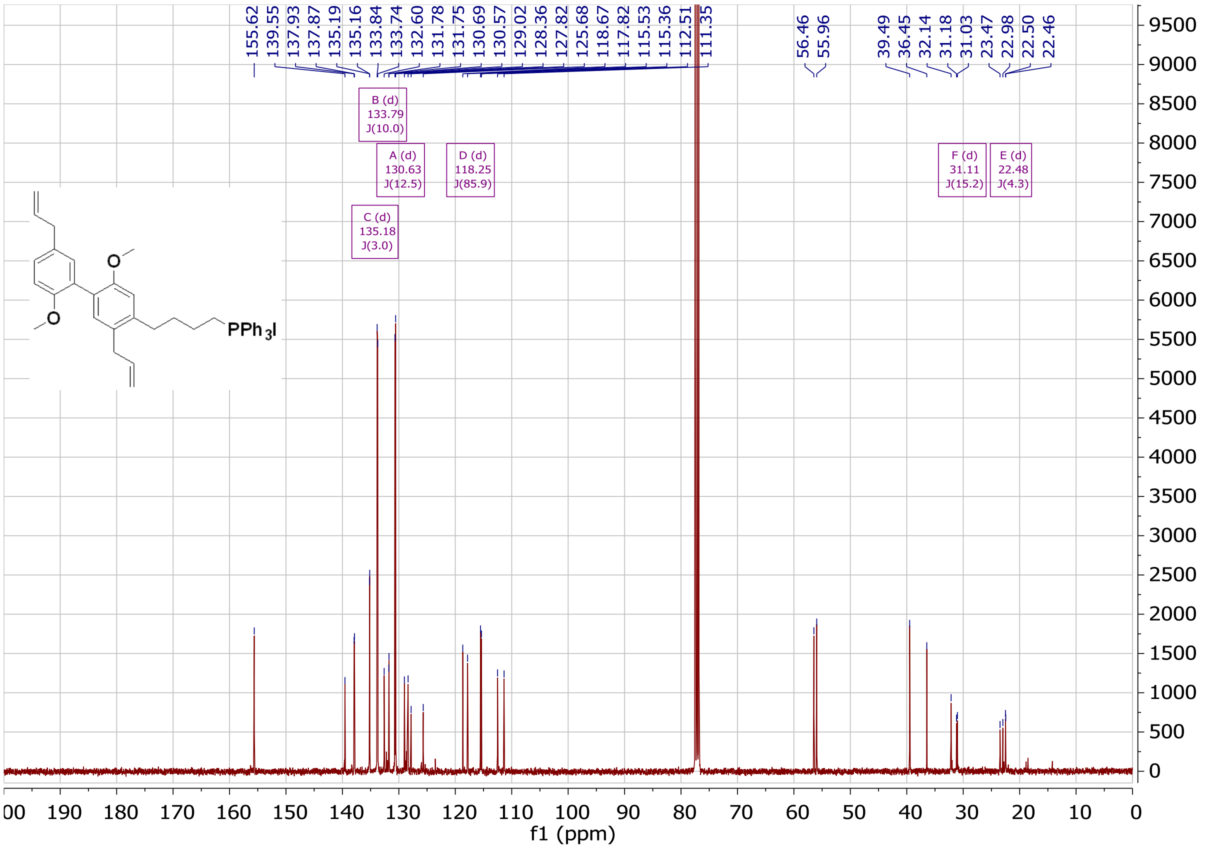

155.62 139.55 137.93 137.87 135.19 135.16 133.84 133.74 132.60 131.78 131.75 130.69 130.57 129.02 128.36 127.82 125.68 118.67 117.82 115.53 115.36 112.51 111.35
56.46 55.96
39.49 36.45 32.14 31.18 31.03 23.47 22.98 22.50 22.46
B (d) 133.79 J(10.0)
A (d) 130.63 J(12.5)
D (d) 118.25 J(85.9)
C (d) 135.18 J(3.0)
F (d) 31.11 J(15.2)
E (d) 22.48 J(4.3)
PPh3I
f1 (ppm)

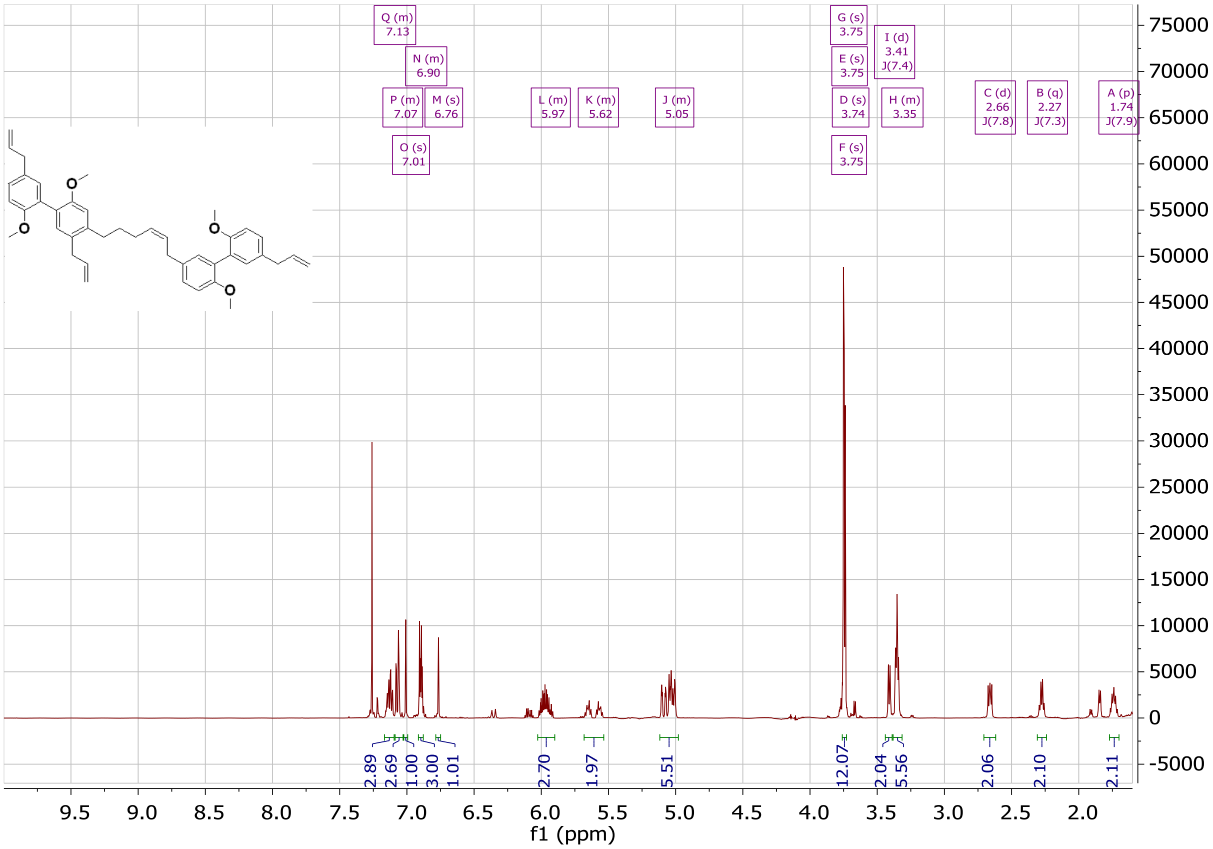

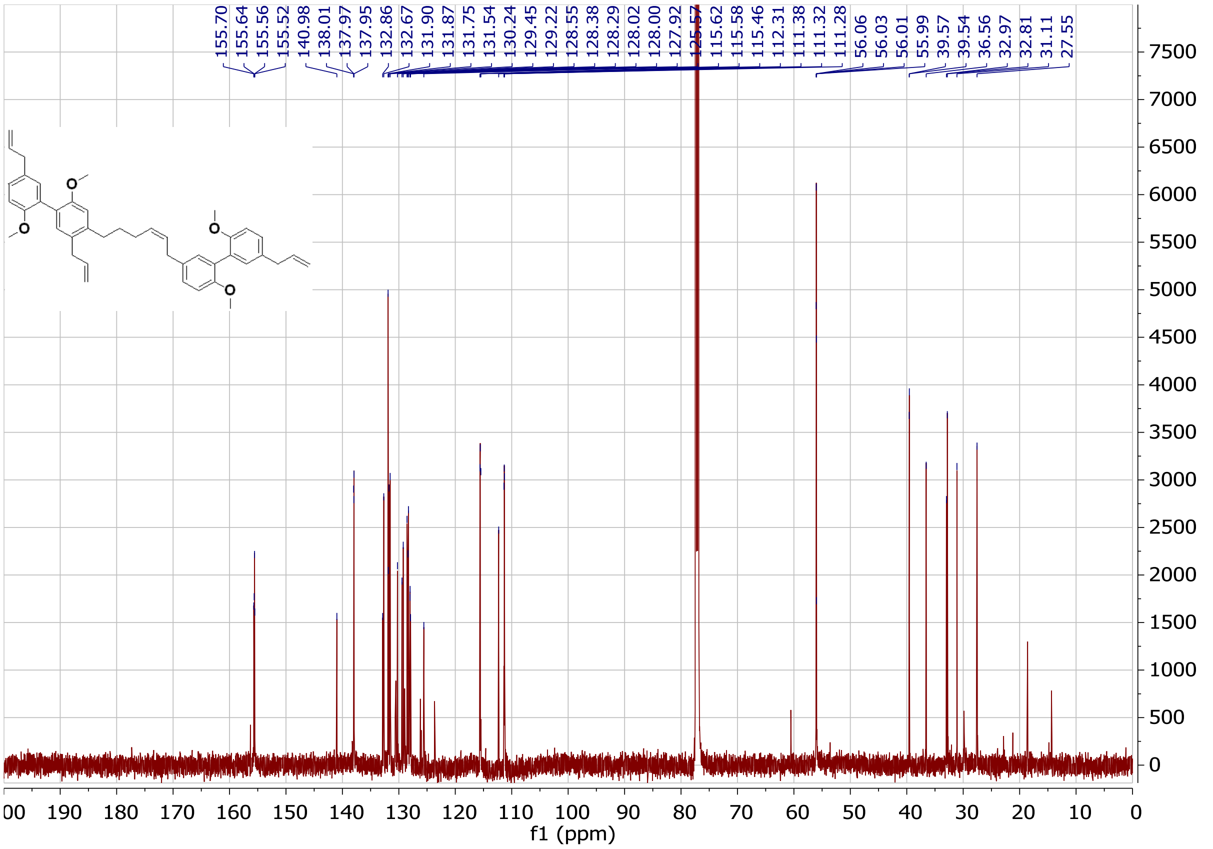

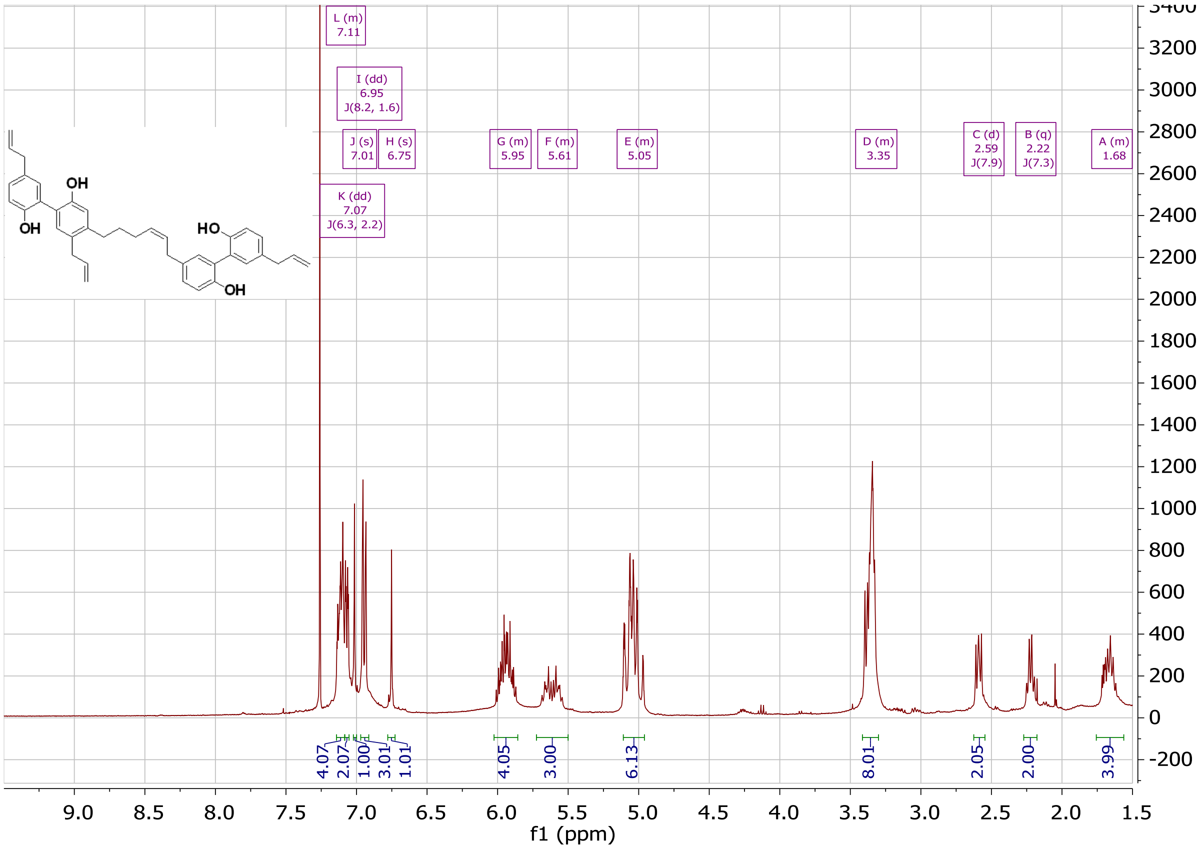
L (m) 7.11
I (dd) 6.95 J(8.2, 1.6)
J (s) 7.01
H (s) 6.75
K (dd) 7.07 J(6.3, 2.2)
G (m) 5.95
F (m) 5.61
E (m) 5.05
D (m) 3.35
C (d) 2.59 J(7.9)
B (q) 2.22 J(7.3)
A (m) 1.68
OH
OH
HO
OH
4.07
2.07
1.00
3.01
1.01
4.05
3.00
6.13
8.01
2.05
2.00
3.99
f1 (ppm)

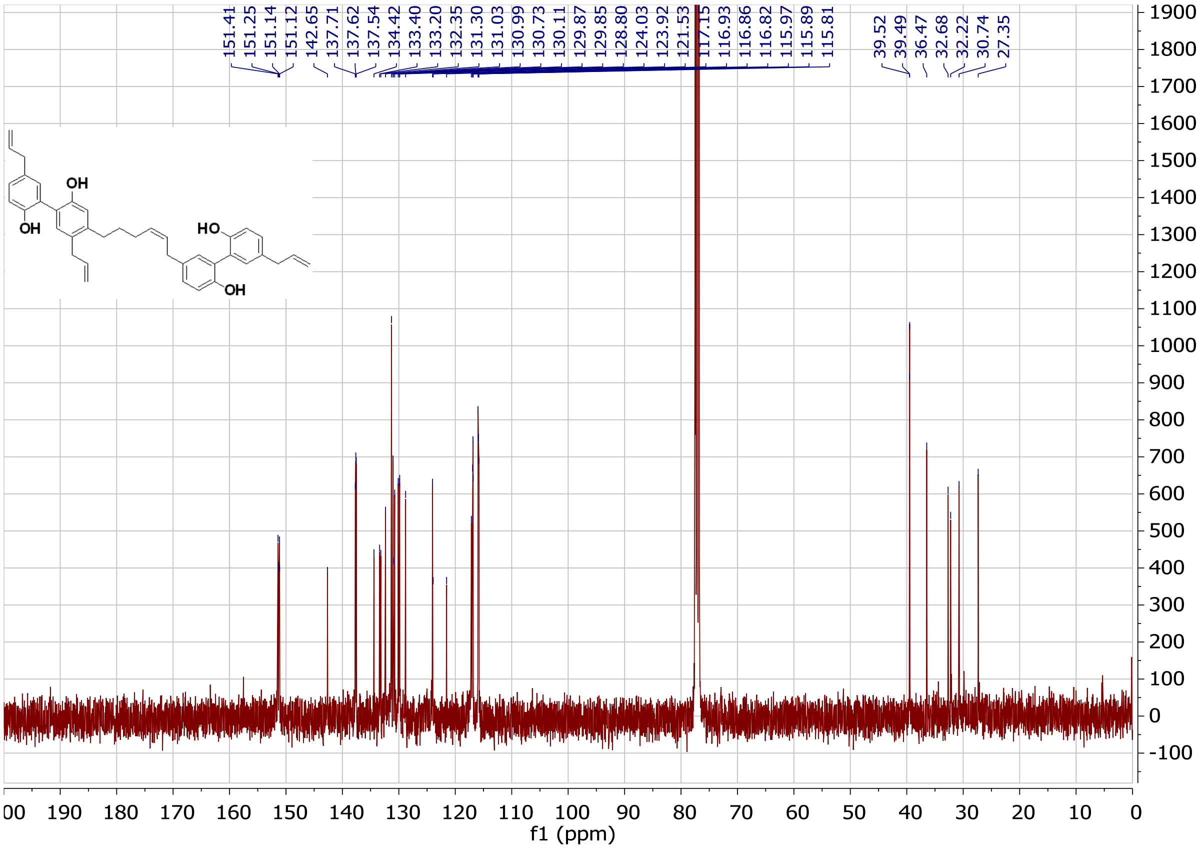
151.41
151.25
151.14
151.12
142.65
137.71
137.62
137.54
134.42
133.40
133.20
132.35
131.30
131.03
130.99
130.73
130.11
129.87
129.85
128.80
124.03
123.92
121.53
117.15
116.93
116.82
115.97
115.89
115.81
39.52
39.49
36.47
32.68
32.22
30.74
27.35
OH
OH
HO
OH
f1 (ppm)

## Assessment of cytotoxicity

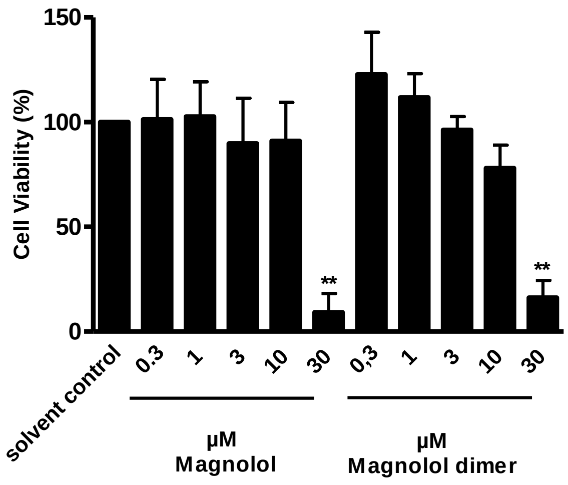


**Supplementary Figure 1**. Influence of increasing concentrations of magnolol or magnolol dimer (**1**) on cell viability assessed by the resazurin conversion assay in HEK293 cells. HEK293 cells were incubated for 24 hours with increasing concentrations of magnolol or magnolol dimer and resazurin conversion was measured after 4 hours. Results are expressed as % cell viability compared to the solvent control DMSO (0.1%). Data are expressed as means ± SD of three independent experiments performed in quadruplicate. (One-way ANOVA, Dunnett´s test, p<0.01**).

A resazurin conversion assay[4] was performed to assess possible cytotoxic effects of magnolol dimer (**1**). Resazurin is a blue non-fluorescent agent that can be chemically reduced to the fluorescent pink resofurin. Conversion of this non-fluorescent agent to resofurin depends on redox equivalents provided by cellular metabolism. Thus, the conversion (fluorescence signal) is direct proportional to the number of viable cells and thus to cell viability. HEK293 cells (ATCC, Manassas, VA) were seeded in 96-well plates at a density of 40,000 cells per well and incubated for 24 hours in DMEM supplemented with 2 mM glutamine, 100 U/ml benzylpenicillin, 100 µg/ml streptomycin, and 10% fetal bovine serum. Next day, cells were incubated with increasing concentrations of the indicated compounds (magnolol or magnolol dimer (**1**)) dissolved in DMSO (0.1%). After 24 hours of incubation with the test compounds cells were washed again in PBS and then incubated with 10 µg/mL resazurin

solution in PBS. After 4 hours plates were read on a Tecan GeniosPro plate reader using an excitation wavelength of 535 nm and an emission wavelength of 580 nm.

## Adipocyte differentiation in 3T3-L1 cells

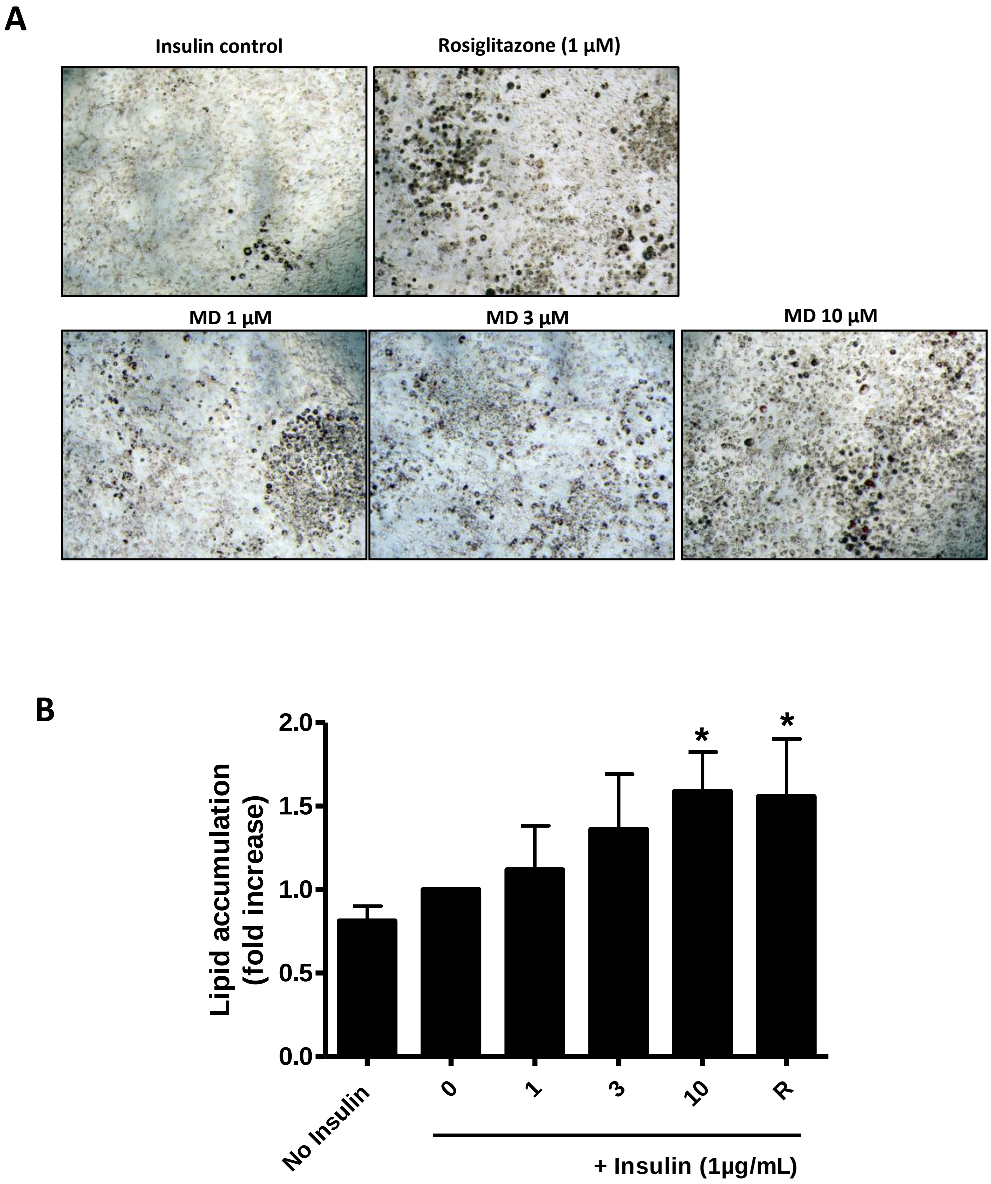


**Supplementary Figure 2**. Adipocyte differentiation in 3T3-L1 pre-adipocytes in response to magnolol dimer (**1**). 3T3-L1 cells were incubated in medium containing insulin (1 µg/mL) for 8 days with or without increasing concentrations of magnolol dimer (**1**) (MD, 1, 3 and 10 µM). Rosiglitazone (R, 1 µM) was used as positive control. The medium was exchanged every second day. At day 8, cells were fixed in 10% formaldehyde, stained with Oil Red O and photographed (A). Oil Red O was then

solubilized and quantified at 550 nm using a plate reader (B). Lipid accumulation is expressed as fold increase compared to the insulin/solvent control (1 µg/mL insulin in 0.1 % DMSO) and is expressed as means ± SD of three independent experiments (One-way ANOVA, Bonfferoni post test, $p<0.05$ *).

3T3-L1 pre-adipocytes were subcultivated in DMEM containing 10% NBS (supplemented with 2 mM glutamine, 100 U/ml benzylpenicillin, 100 µg/ml streptomycin). Cells were grown until confluency in 12-well plates. Two days later cells were treated with DMEM containing 10% FBS (no insulin control) or DMEM/10% FBS/1µg/ml insulin (differentiation medium) containing increasing concentrations of magnolol dimer (1, 3 and 10 µM). Medium was exchanged every second day. After eight days, lipid accumulation (as a measure of adipogenic differentiation) was determined by Oil Red O staining as described previously.[5] For this, cells were fixed in 10% formaldehyde for 1 h and stained with Oil Red O for 10 minutes. After washing off the excessive dye, photos were taken, and bound dye was solubilized with 100% isopropanol and photometrically quantified at 550 nm (Tecan Sunrise).

# References


1. Roberts, J. C.; Pincock, J. A., The photochemistry of 1-(3,5-dimethoxyphenyl)-2-(4-methoxyphenyl)ethyl ethanoate in alcohol solvents: A search for carbocation rearrangements. *Canadian Journal of Chemistry* **2003,** 81, (6), 709-722.
2. Yan, C.-S.; Peng, Y.; Xu, X.-B.; Wang, Y.-W., Nickel-Mediated Inter- and Intramolecular Reductive Cross-Coupling of Unactivated Alkyl Bromides and Aryl Iodides at Room Temperature. *Chemistry - A European Journal* **2012,** 18, (19), 6039-6048, S6039/1-S6039/224.
3. Stanetty, P.; Mihovilovic, M. D., Half-Lives of Organolithium Reagents in Common Ethereal Solvents. *Journal of Organic Chemistry* **1997,** 62, (5), 1514-1515.
4. O'Brien, J.; Wilson, I.; Orton, T.; Pognan, F., Investigation of the Alamar Blue (resazurin) fluorescent dye for the assessment of mammalian cell cytotoxicity. *Eur J Biochem* **2000**, 267, 5421-6.
5. Fakhrudin, N.; Ladurner, A.; Atanasov, A.G.; Heiss, E.H.; Baumgartner, L.; Markt, P.; Schuster, D.; Ellmerer, E.P.; Wolber, G.; Rollinger, J.M.; Stuppner, H.; Dirsch V.M. Computer-aided discovery, validation, and mechanistic characterization of novel neolignan activators of peroxisome proliferator-activated receptor gamma. *Mol Pharmacol* **2010**, 77,559-66.
